# Complete re-sequencing of a 2Mb topological domain encompassing the FTO/IRXB genes identifies a novel obesity-associated region upstream of IRX5

**DOI:** 10.1186/s13073-015-0250-3

**Published:** 2015-12-07

**Authors:** Lilian E. Hunt, Boris Noyvert, Leena Bhaw-Rosun, Abdul K. Sesay, Lavinia Paternoster, Ellen A. Nohr, George Davey Smith, Niels Tommerup, Thorkild I. A. Sørensen, Greg Elgar

**Affiliations:** The Francis Crick Institute, Mill Hill Laboratory, The Ridgeway, Mill Hill, London, NW7 1AA UK; Genomics Facility, The Francis Crick Institute, Mill Hill Laboratory, The Ridgeway, Mill Hill, London, NW7 1AA UK; MRC Integrative Epidemiology Unit (IEU) at the University of Bristol, University of Bristol, Bristol, UK; Research Unit for Gynecology and Obstetrics, Institute of Clinical Research, University of Southern Denmark, Odense, Denmark; Willhelm Johannsen Centre for Functional Genome Research, Department of Cellular and Molecular Medicine, The Faculty of Health Sciences, The University of Copenhagen, Blegdamsvej 3B, DK-2200 Copenhagen N, Denmark; The Novo Nordisk Foundation Centre for Basic Metabolic Research, Section on Metabolic genetics, The Faculty of Health and Medical Sciences, University of Copenhagen, DK2100 Copenhagen Ø, Denmark; Institute of Preventive Medicine, Bispebjerg and Frederiksberg Hospitals, The Capital Region, DK2000 Frederiksberg, Denmark

## Abstract

**Background:**

Association studies have identified a number of loci that contribute to an increased body mass index (BMI), the strongest of which is in the first intron of the FTO gene on human chromosome 16q12.2. However, this region is both non-coding and under strong linkage disequilibrium, making it recalcitrant to functional interpretation. Furthermore, the FTO gene is located within a complex cis-regulatory landscape defined by a topologically associated domain that includes the IRXB gene cluster, a trio of developmental regulators. Consequently, at least three genes in this interval have been implicated in the aetiology of obesity.

**Methods:**

Here, we sequence a 2 Mb region encompassing the FTO, RPGRIP1L and IRXB cluster genes in 284 individuals from a well-characterised study group of Danish men containing extremely overweight young adults and controls. We further replicate our findings both in an expanded male cohort and an independent female study group. Finally, we compare our variant data with a previous study describing IRX3 and FTO interactions in this region.

**Results:**

We obtain deep coverage across the entire region, allowing accurate and unequivocal determination of almost every single nucleotide polymorphism and short insertion/deletion. As well as confirming previous findings across the interval, we identify a further novel age-dependent association upstream of IRX5 that imposes a similar burden on BMI to the FTO locus.

**Conclusions:**

Our findings are consistent with the hypothesis that chromatin architectures play a role in regulating gene expression levels across topological domains while our targeted sequence approach represents a widely applicable methodology for high-resolution analysis of regional variation across candidate genomic loci.

**Electronic supplementary material:**

The online version of this article (doi:10.1186/s13073-015-0250-3) contains supplementary material, which is available to authorized users.

## Background

Previous genome-wide association studies (GWAS) have consistently identified single nucleotide polymorphisms (SNPs) associated with obesity located within the first intron of the *FTO* gene on human chromosome 16q12.2 [1–3]. Findings from these studies have been confirmed in meta-analyses wherein the associated SNPs are in strong linkage disequilibrium (LD) with one another. The strongest association is found for SNP rs1121980:C > T with an odds ratio of 1.66 among 929 Caucasians [2]. This variant is in LD with a number of other SNPs (r^2^ ≥ 0.88 for all), including rs9939609:T > A, which has been the most extensively genotyped. The rs9939609 risk allele (A) has an odds ratio itself of 1.34 for heterozygotes and 1.55 for homozygotes [4]. This association has also been identified for type 2 diabetes (T2D); however when adjusting for body mass index (BMI), the T2D association is lost suggesting that this association is a secondary effect of BMI [1].

The association of obesity with rs9939609:T > A has been replicated in many independent study groups across a range of different ethnicities [5–10]. However, the degree of linkage disequilibrium across the entire intron 1 of FTO has prevented a single potentially functional SNP from being identified, although trans-ethnic comparison has permitted a degree of fine mapping of the region [11]. The LD region identified in the HapMap Phase II data spans about 50 kb, covering part of the first intron of *FTO*, the second exon and a small portion of the second intron [12]. Despite this, coding SNPs in the second exon of *FTO* have not been found to follow the same association patterns.

As a result of the persistent association with obesity in this region, the function of the nearest gene, *FTO*, has been under close scrutiny. FTO is a ubiquitously expressed *N*6-methyladenosine demethylase [13], yet there are conflicting data and models of how changes in *FTO* expression might affect function and phenotype. Mouse models have been informative; knockdown of *FTO* in mice results in reduced fat mass, suggesting that the susceptibility to obesity could be through over-expression of *FTO* [14]. A further mouse *FTO* knockout has been described generated through replacement of exons 2 and 3 with a neomycin STOP cassette [15]. This mouse exhibits growth retardation from postnatal day 2 onwards although it also shows a broader range of phenotypes including higher postnatal death. It supports the hypothesis that FTO is involved in energy metabolism and body weight regulation as the knockout mice show a reduction in adipose tissue and increased energy expenditure. However, eQTL analyses examining the links between the associated SNPs and the expression levels of *FTO* have not to date identified a clear and direct correlation [16–18].

A few hundred bases upstream of *FTO*, and transcribed in the opposite direction, is the *RPGRIP1L* gene. As a result of its proximity to the LD region, the function of this gene has also been closely examined on the premise that non-coding SNPs might affect the regulatory landscape acting in *cis* on this nearby gene. Some evidence to this effect has been reported [19] and *Rpgrip1l*^*+/−*^ mouse models gain weight more rapidly than their wild-type litter mates, as well as exhibiting increased energy intake and increased adiposity [20].

More recently, chromosome conformation capture (3C) approaches have demonstrated that longer-range interactions occur across this region acting at both the *FTO* and *IRX3* gene promoters [21] although the concept of long-range regulation in this region has been speculated upon previously [22]. These studies point to *IRX3* as a further potential candidate gene that might interact with the associated SNPs in the first intron of FTO. In the paralogous IRXA cluster (encompassing *IRX1*, *IRX2* and *IRX4* at a separate genomic location on chromosome 5), there has already been some enhancer analysis that suggests co-regulation of all three genes [23]. Therefore it might be that a similar pattern of distal *cis*-regulation operates at this obesity-associated locus.

Further evidence to support this comes from the analysis of topologically associated domain (TAD) structure in mammalian genomes. Data from embryonic stem cells identify a TAD of approximately 2 Mb that neatly encompasses the IRXB cluster, *FTO* and *RPGRIP1L* genes (chr16:53,562,500-55,442,500) [24]. Hence perturbation of the transcriptional architecture within this region during development could potentially impact upon any or all of these genes, and lead to an altered BMI phenotype.

Finally, it is of note that this region contains hundreds of deeply conserved non-coding elements (CNEs), sequences implicated in the long-range cis-regulation of genes during development, including the IRX genes. Variants in such sequences might result in altered gene expression profiles across the region. Interestingly, the locations of CNEs at the IRXB cluster span from 53.56 to 55.48 Mb, in remarkably close agreement to the boundaries of the TAD [24, 25].

Here, using custom enrichment, we generate and analyse the complete sequence of 284 Danish males homozygous at rs9939609 across the 2 Mb TAD region. The resulting deep and comprehensive coverage allows us to identify over 14,000 SNPs and short indels permitting the precise and complete construction of haplotypes without the need for imputation. The use of homozygotes for the *FTO* LD region facilitates the downstream analysis of haplotypes. We identify a novel association that implicates the *IRX5* gene region in obesity and compare our results with previously derived interaction data for the region. We then replicate our findings in an expanded male cohort and in a separate female study group using accurate imputation calls, and identify an age dependent association, consistent with previous studies [26, 27]. Finally, our study provides a high quality, single base resolution resource for further study into the complex genetics of obesity across human chromosome 16q12.2, and a general methodology for targeted sequencing and analysis of variation across large genomic regions in general.

## Results

### Strategy and study group

We employed a custom in-solution hybridisation approach to capture and completely sequence a 2 Mb region of chromosome 16 encompassing the *RPGRIP1L*, *FTO* and *IRX3*, *5* and *6* genes from 288 Danish men, previously genotyped as homozygous at rs9939609 (A/A or T/T) [28]. The region (53.5 to 55.5 Mb) was specifically selected to encompass a TAD defined in embryonic stem cells (53.56–55.44 Mb) (24). Our study group comprises 126 cases with a BMI of ≥31.0 kg/m^2^ and 162 control samples (Additional file 1: Table S1). They originate from two larger series of men selected from the study population of Danish men (n = 362,200) examined at mandatory draft board assessment during the years 1943 through 1977 [28]. The case set represents all men with a BMI ≥31.0 kg/m^2^ at initial assessment, corresponding to those above the approximately 99.5 percentile, whereas the control group consists of a randomly selected 1 % of all men in the original study population and is thus representative of the underlying population’s distribution of BMI values. The case group and half of the control group have been used in several follow-up studies including one in 1998–2000 where additional blood sampling allowed extraction of high quality DNA [28–33]. As a result of this sampling design, our study group has a bi-modal distribution of BMI values and enrichment for homozygosity across the LD region encompassing the obesity-associating SNPs. The average BMI for the controls is 21.5 compared to 33.2 for the cases (Table 1). BMI values are calculated from the original draft board assessment. The rs9939609:T > A (risk) allele was present in our study group at 41.9 %. In 1000 Genomes Project (1KG) data, both the Finnish (FIN) and British (GBR) allele frequency (AF) of the minor allele is 39.3 % [34]. Therefore, despite our enrichment for homozygosity, we have a similar representation of the risk allele compared to the general population. The study group also maintains the relative proportions of T/T to A/A individuals (1.9:1 in controls and 0.9:1 in cases) found in the larger case and control group from which our individuals are derived [28].

**Table 1:** Study group details

	rs9939609					
	T/T (%)	A/A (%)	Total (%)	Average BMI	Variance (95 % CI)	Standard error from mean
Controls. BMI <31 kg/m^2^	106 (37.3)	55 (19.4)	161 (56.7)	21.5	±0.4	4.3
Cases. BMI ≥31 kg/m^2^	59 (20.8)	64 (22.5)	123 (43.3)	33.2	±0.5	5.6
Total	165 (58.1)	119 (41.9)	284 (100)	26.5	±0.7	7.1

### Sequencing and variant calling

We used 96-plex indexing to construct custom libraries for 288 samples. We generated 1.66 billion paired end reads from these libraries for a total of 166 Gb of sequence. Approximately 75 % of reads map back uniquely to the 2 Mb region of interest (chr16:53,500,000-55,500,000) giving an average of 4 million reads per sample (200-fold coverage). Our sequencing identified one genotyping error (a genotyped A/A individual that was actually A/T), one sample failed to run, and two samples (1 case T/T, 1 control T/T) were of low coverage and had missing genotypes for more than 50 % of variants. These were removed from subsequent analyses resulting in a final set of 284 samples (161 controls and 123 cases). As expected, coverage varied extensively both between samples and across the region. Nevertheless, 277 samples have greater than 10-fold coverage across at least 90 % of the region (Additional file 2: Figure S1) allowing comprehensive, single base resolution analysis and unequivocal variant calling.

We developed an in-house variant calling algorithm ‘TidyVar’ (methods – B. Noyvert and G. Elgar, manuscript in preparation). The algorithm is fundamentally different from that of commonly used variant calling software GATK [35]. TidyVar can be accurately deployed across any region of DNA of any size and from any species. Across the two-megabase interval, 14,101 variants passed quality control, of which 13,373 are simple (bi-allelic) and 728 are ‘complex’, in that they have more than one non-reference allele. Of the 13,373 simple variants, 12,392 are SNPs and 981 are indels. Fifty-nine percent of these variants are identically catalogued in the phase 1 release of 1KG project data [34] and 74 % are identically catalogued in dbSNP build 142 [36]. On average, each individual has 2,869 variants across the region (ranging from 2,178 to 3,377).

**Fig. 1:**
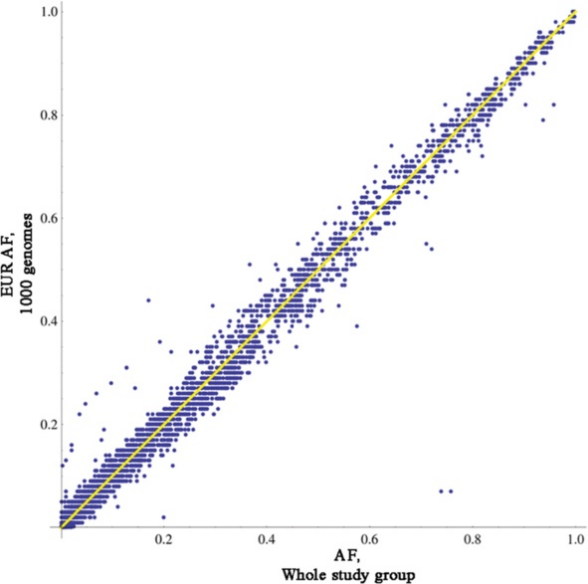
Variant frequencies across the 2 Mb interval. The allele frequency of each variant in our study group is plotted against its frequency in European populations from the 1,000 Genomes Project. Only variants identified in both sets of data in the same format are directly compared (n = 8338). Two SNPs give very high values in our study group but low values in the Europeans population; these are artefacts caused by simple sequence repeats

We compared minor allele frequency (MAF) for those bi-allelic SNPs present in both our whole study group and the 1KG project (Fig. 1). Reassuringly, the two datasets correlate very closely, demonstrating that despite selecting only homozygotes, the fact that we frequency matched rs9939609:T > A with the general European population results in a broadly representative set of variant frequencies.

We looked for copy-number variations (CNV) and found that four individuals, all of whom are obese, have an 11.6 Kb deletion (esv2659911) on one allele at chr16:54,408,013-54,419,653. The probability that four randomly picked samples are cases is significant at the 5 % level (*P* value = 0.034). However, based on this small study alone, we are not able to firmly establish if this deletion is associated with obesity in the general population.

### Distribution of variants across constrained sequences

Within the 2 Mb interval sequenced in this study, 225 conserved CNEs are highly conserved between mammals and fish (CONDOR [25]) covering a total of more than 25 kb. In addition, there is 17.2 kb of coding sequence across the region. We examined the number and distribution of SNPs in these different classes of constrained DNA (Table 2, Additional file 3: Figure S2, Additional file 4: Figure S3). As expected, there is a lower density of SNPs in coding sequences and to a lesser extent in CNEs, than in the remainder of the non-coding DNA across the region. SNPs in coding sequences and CNEs also have lower mean MAFs than general non-coding DNA, reflecting an excess of rare variants (Additional file 5: Figure S4). These data reflect differing levels of functional constraint at these sites. However, the number of variants per individual does not differ significantly between cases and controls in any class of sequence.

**Table 2:** Variant summary data for chr16q12.2 classified by functional region and BMI status

Region	Size of region (kb)	Number of variant locations	Variant locations per Kb	Mean MAF	Average number variants per individual	Average number non-ref alleles per individual
CNEs	25.1	Cases 117	4.66	0.118	20.03	27.52
		Controls 109	4.34	0.127	19.94	27.60
		Total 141	5.61	0.098	19.98	27.57
Coding	17.2	Cases 38	2.21	0.075	4.61	5.37
		Controls 56	3.26	0.051	4.75	5.40
		Total 70	4.07	0.041	4.69	5.39
Non-coding	1,957.7	Cases 11014	5.51	0.181	2853	3916
		Controls 11826	5.91	0.167	2836	3892
		Total 13980	6.94	0.142	2843	3902

### Haplotype analysis

**Fig. 2:**
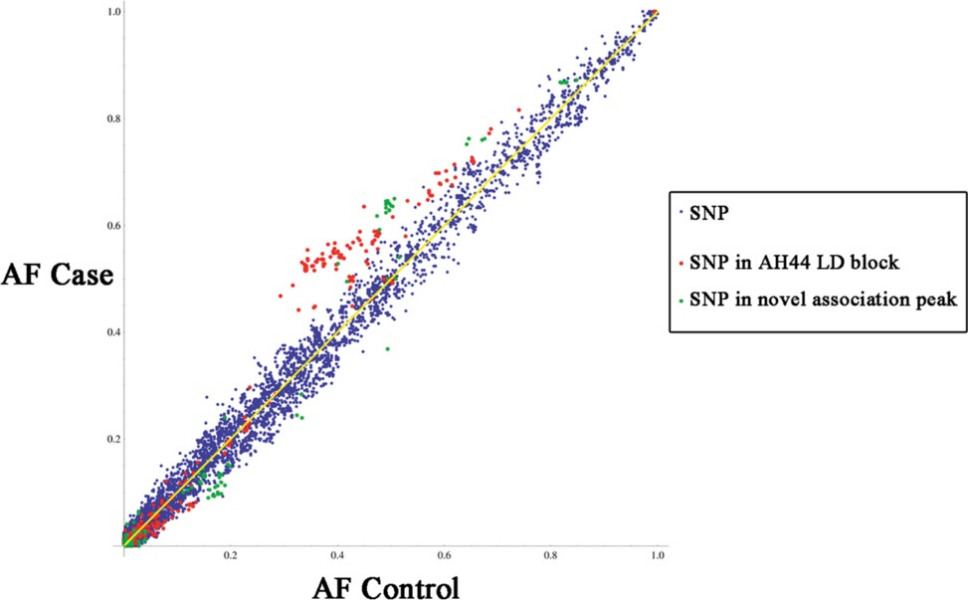
Allele frequencies for each variant across the 2 Mb interval compared between controls and cases. Variants within the AH44 LD block are in red and variants in the second association region upstream of IRX5 are in green

Haplotype analysis of the entire region (using pairwise comparison of SNPs up to 500 kb apart) permits the identification of blocks with high LD (Additional file 6: Figure S5), the most notable of which is the previously identified 44 kb region (chr16:53,799,296-53,843,533) in the first intron of the FTO gene containing rs9939609. Three distinct haplotypes persist across this interval and comprise 63.5 % of all haplotypes across the region. The first two (29.3 % and 12.5 %) differ by just one SNP (rs113191842:A > G) and account for all the rs9939609 A/A individuals (known henceforth as haplotype AH44). While the more common of these two haplotypes strongly associates with the obesity case group (Fig. 2) as expected (*P* = 1 × 10^-4^), the second does not (*P* = 0.383) although this might simply reflect a lack of statistical power due to its low frequency in the study group overall. The third common haplotype (21.7 %) is found only in T/T individuals, but does not show a significant association with either case or control (*P* = 0.086) group. Additional file 7: Table S2 describes all the other haplotype blocks with associations to the case or control group with a frequency of >0.05. Due to the number of individuals sequenced in this study, we have focused on the two regions showing the clearest and most strongly associating variants. There are several other LD blocks containing haplotypes that also associate with either case or control outside of these regions, however further sequencing would be needed to establish any association of these blocks in the general population.

### The AH44 haplotype

The obesity-associated haplotype, along with its almost identical sub-haplotype (referred to collectively henceforth as AH44) has a clear and distinct pattern of variation across its length when compared to other haplotypes for the same region. This haplotype encompasses many of the obesity-associated SNPs that have been identified by various GWAS studies [37]. From our in depth analysis, 114/122 highly polymorphic SNPs (MAF >0.35) spanning 53,798,523 to 53,848,561 (50.038 kb) are in complete LD with rs9939609 in all A/A individuals in our study group. Of the remaining 8/122 SNPs, seven are uniquely heterozygous in the same individual and the final SNP also occurs just once. While these common SNPs are essentially in complete LD across the 44 kb AH44 haplotype, there are a number of rarer variants across the region that are not in LD, indicating that while the common variants are retained, there are in fact numerous sub-haplotypes that contribute to AH44. The most frequent of these rarer alleles that separates the two AH44 haplotypes (see above), rs113191842:A > G at 53,817,318 (just over 3 kb from rs9939609:T > A), is only present in AH44 but occurs at a frequency of 0.28 within this population. A further 26 non-unique and 21 unique variants are present within the AH44 haplotype individuals, while just one of these (rs16952522:C > G) is shared with the non-AH44 haplotype in T/T individuals.

### Identification of a novel region associated with BMI in our study group

**Fig. 3:**
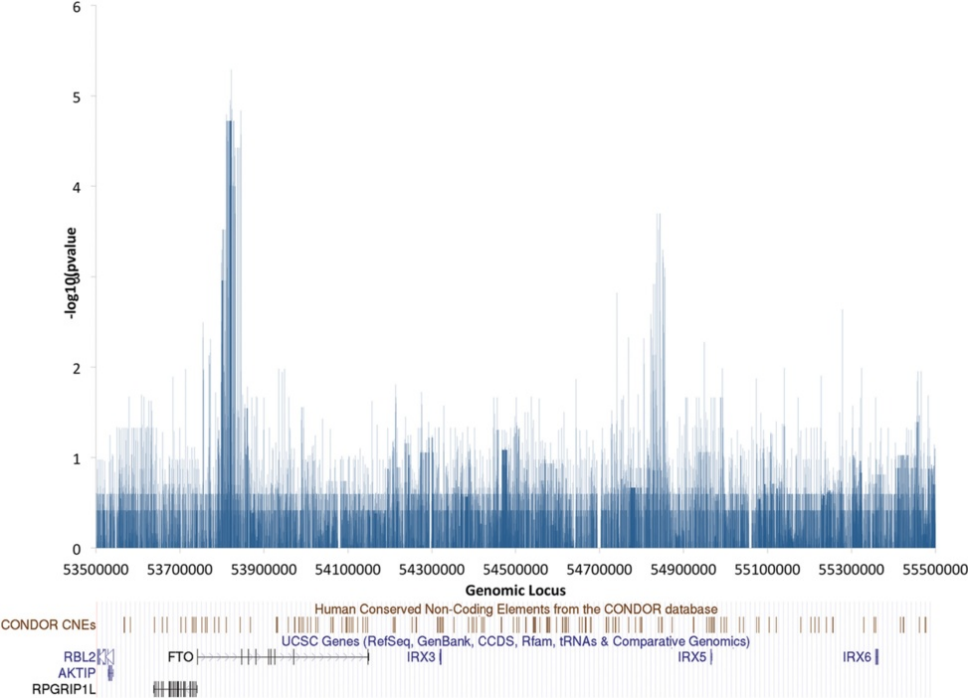
Association of SNPs in cases vs. controls. Minus log10(*P* value) of Case/Control association for each SNP across the 2 Mb interval generated from Haploview [38] and represented by vertical blue lines. The first peak (at 53.82 Mb) in the intron of FTO shows the known association at rs9939609:T > A and reflects the strong linkage disequilibrium across that region. The second peak (54.84 Mb) indicates a novel associated region upstream of IRX5

**Fig. 4:**
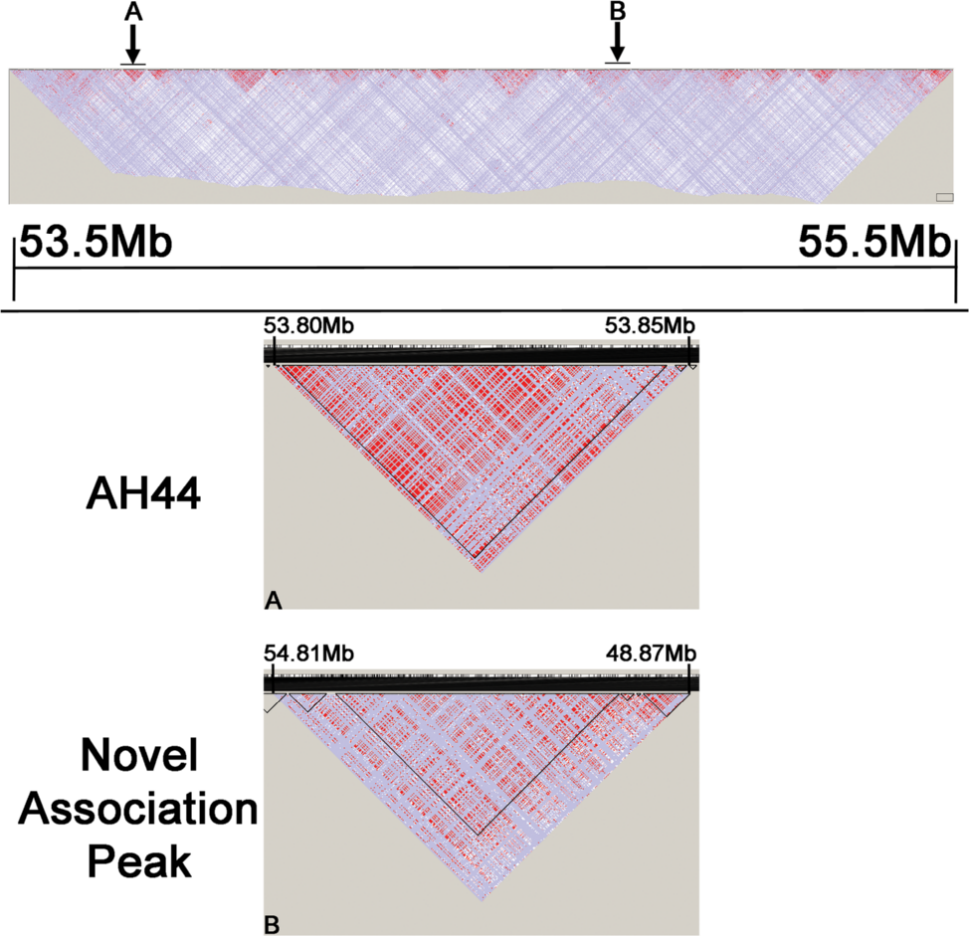
Linkage disequilibrium across the two associated regions. Mountain plots generated by Haploview [38] for (**a**) rs9939609:T > A associated region chr16:53,797,908-53,846,168 (48.3 kb) and (**b**) the novel associated region at chr16:54,812,014-54,865,446 (53.4 kb), across our complete study group. Bright red represents D’ = 1 and LOD ≥ 2 (complete dependence and strong evidence for linkage); shades of pink/red represent D’ < 1 and LOD ≥ 2; blue represents D’ = 1 and LOD < 2; white represents D’ < 1 and LOD < 2(low dependency and poor evidence for linkage). The full plot of the region sequenced is shown for reference (chr16:53500000-55500000)

We used Haploview [38] to compare the frequency of every SNP across the 2 Mb interval between cases and controls and to calculate the case-control allelic association *P* values (Fig. 3 and methods). The known LD region in the first intron of *FTO* (chr16:53,797,908-53,846,168) is clearly defined (Fig. 4a). In addition, we also observe a second peak of association approximately 1 Mb away that consists of a cluster of SNPs upstream of the *IRX5* gene (16:54,820,000-54,860,000). Critically, this case specific association is independent of risk allele rs9939609 (Additional file 6: Figure S5 and Additional file 8: Figure S6). Furthermore, random shuffling of the cases and controls results in loss of any comparable signal across the region (Additional file 9: Figure S7). The non-coding region encompasses four linkage disequilibrium blocks, the largest of which is 38 kb in size (Fig. 4b). Within this LD block, there are several haplotypes identified by Haploview.

The strongest obesity associating haplotype (*P* value = 0.002) occurs at a frequency of 0.49 in the case group and 0.36 in controls. No other haplotype in this LD block has a total frequency above 0.13 suggesting that the associating haplotype is more robust in its entirety than the multiple non-associating haplotypes. The 38 kb associating region encompasses 213 SNPs that we have identified in our sequencing, 78 of which are tagged by Haploview for use in haplotyping. Thirty-five SNPs within this LD block have an association *P* value <0.05. The lowest 10 % of *P* values for SNPs in this region (n = 21) range from 0.0002 to 0.0073. The three highest associating SNPs in this second peak (*P* = 0.0002) are in complete LD within 7 kb of each other in all but two individuals. These SNPs (rs7186407:A > T, rs12598453:C > G and rs12596270:A > G, hg19 coordinates chr16: 54837068, 54843731, 54843981, respectively) are present at a frequency of 0.491 in controls and 0.646 in cases (0.56 in whole study group), whereas they have a wide range of derived allele frequencies in different populations in the 1KG Project, with values as low as 0.0225 to 0.036 in Japanese and Chinese populations, to greater than 0.5 in all European populations. Individuals in the study group who have neither risk region allele rs9939609:T > A (from known region) nor rs12598453:C > G from our novel region have a mean BMI of 23.86, whereas individuals homozygous for either risk region have significantly higher mean BMIs of 27.96 (Mann-Whitney *P* value = 0.0062) and 27.60 (*P* value = 0.0088), respectively (28.90 if homozygous for both (*P* value = 0.00067)). Thus, both regions have a similar association with BMI.

### Multiple testing correction

The *P* values presented in the previous section are not corrected for multiple testing. A naïve Bonferroni correction for 14 k variants would give a *P* value threshold significance of 3.5 × 10^−6^ (=0.05/14000) when controlling the family wise error rate (FWER) at the 5 % level. However, since the variants are not independent the above correction is overly conservative. Indeed variants belonging to the same linkage disequilibrium blocks have a strong positive correlation (consider that we identify multiple associating variants across both the known, and our novel, regions). It is therefore more appropriate to use the number of LD blocks (n = 226) identified by Haploview to estimate the corrected *P* value threshold. This then becomes 0.05/226 = 2.2 × 10^−4^. Only the known LD region in the first intron of *FTO* and the second peak of association identified above pass this threshold (Fig. 3), guiding us to focus on these two regions only.

Since there is no exact definition of an LD block the above multiple testing correction by the number of LD blocks may be underestimated. This is why we decided to control for FWER by permuting the set of obese and control labels. This was achieved by a 100,000-permutation test in Haploview for the full set of sequenced variants across the 2 Mb in our cohort of 284 men. The individual SNPs in the second peak of association have corrected *P* values >0.05 and therefore do not pass multiple testing correction. This is a reflection of our limited sample size and paradoxically the vast number of variants we identified through complete sequencing of the region. Therefore it was essential that we replicate our findings in other cohorts.

### Replications

In order to validate our findings, we replicated our case-control association tests in two larger cohorts (Table 3). The first (Male GOYA) comprises 1,450 men from the expanded cohort that our study group was initially selected from [31]. The expanded group has imputed SNP data for the three highest associating SNPs (rs7186407:A > T, rs12598453:C > G and rs12596270:A > G) as well as for rs9939609:T > A. The three highest associating SNPs, which are in near perfect LD, were chosen to be representative of the second novel peak of association. We found that in this larger group of young men, all three representative SNPs also associate with the case group, with a *P* value of 0.0054 (Table 3).

**Table 3:** Replication data using SNP rs12598453:C > G as a representative of the three SNPs referred to in the text

Cohort	Number of individuals	Number of controls, cases	AF in cases, controls	*P* value, case-control allelic chi-squared test	BMI averages by genotype: CC, CG, GG
Sequenced men	284	161, 123	0.491, 0.646	0.00021	25.2, 26.03, 28.13
Male GOYA	1,450	785, 665	0.496, 0.547	0.0054	26.51, 26.89, 27.39
GOYA men, younger than 25 years	1,381	749, 632	0.493, 0.551	0.0027	26.45, 26.85, 27.47
Female GOYA	3,908	1948, 1960	0.507, 0.529	0.056	30.00, 30.21, 30.46
GOYA women, younger than 25 years	562	255, 307	0.465, 0.560	0.0014	29.79, 30.67, 32.5
All combined	5,401	2762, 2639	0.503, 0.534	0.0012	29.04, 29.24, 29.63
All combined, younger than 25 years	1,984	1032, 952	0.486, 0.556	0.000011	27.35, 27.85, 28.89

In addition, we replicated our association analysis of the three representative SNPs in a large female Danish cohort (Female GOYA) [31, 39]. We initially looked at the entire cohort of 3,908 women (1,960 extremely overweight and 1,948 control women, total average age of 29.5). In this group, using imputed data for the three representative SNPs, we cannot confidently replicate the second association peak (*P* values >0.05, Table 3). However, in light of previous studies that suggest genetic association to obesity at the FTO locus may be age-dependent [26–28] and because of the lower, narrower age range in our male cohort (mean = 19.9), we examined the role of age in this age-diverse female cohort (range from 16 to 45). We find that the allele frequency of the three SNPs is consistently higher in cases compared with controls only for women aged under 25 years (Additional file 10: Figure S8). In this smaller group of 562 individuals the three SNPs show significant association with obesity (*P* value = 0.0014, Table 3). This is consistent both with previous studies at the FTO locus [26] and with the value found in the larger Male GOYA cohort. If we consider all individuals aged less than 25 years in all cohort groups then the *P* value for the association of the novel peak we found is 1 × 10^−5^ (Additional file 11: Figure S9) confirming that our second novel peak of association can be replicated independently in larger cohorts of the same ethnic background and similar age, regardless of gender.

### IRX3 interactions extend beyond both BMI associated regions

**Fig. 5:**
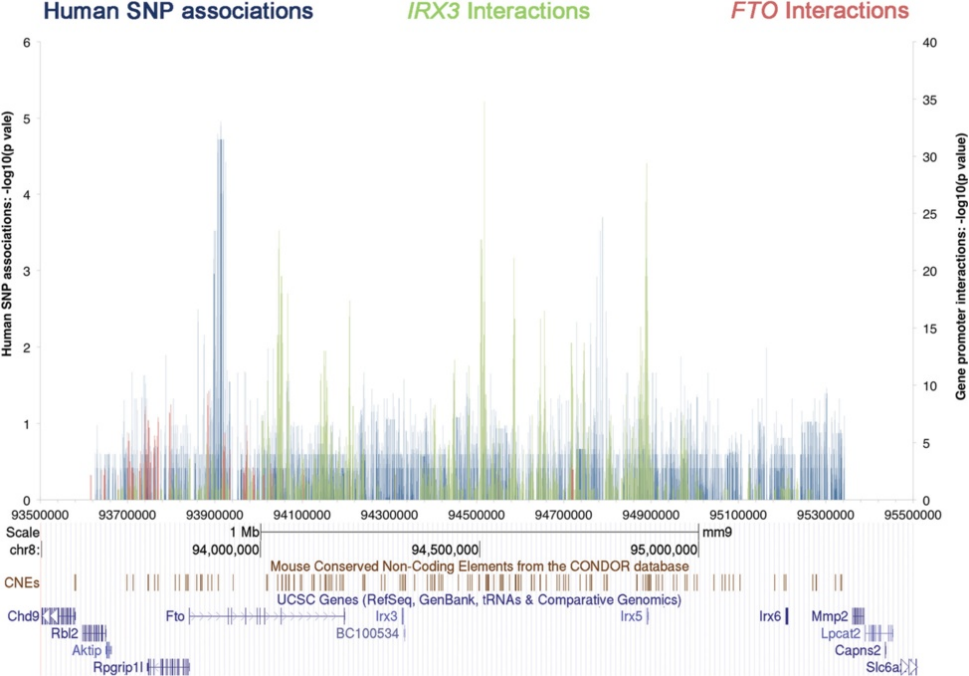
Comparison of association data with previously mapped FTO and IRX3 interactions. Association data for bi-allelic SNPs identified in this study are mapped to the mm9 mouse genome build and represented in blue (methods). The IRX3 (green) and FTO (red) promoter interaction data [21] are shown for the same region allowing a direct comparison of our SNP associations and previously published interaction data

Recently, long-range interactions have been experimentally defined across most of the 16q12.2 region for the FTO and IRX3 genes using chromatin conformation analysis [21]. Comparing the locations of these interactions with those of BMI-associated SNPs might help determine both a mechanism and a role for the SNP regions in the cis-regulation of the FTO or IRX3 genes. Figure 5 shows that while neither the FTO nor the IRX3 promoter-based 4C-seq data correlate strongly with our associated regions, both our regions are within the long-range interaction architecture of IRX3, with particularly strong interactions (both with FTO and IRX3) flanking the associated region upstream of IRX5. Hi-C data from human embryonic stem cells also provides strong evidence that our novel association region upstream of IRX5 plays a role in many interactions across the TAD (Additional file 12: Figure S10), including with the IRX3 and FTO gene regions ([24] and http://yuelab.org/hi-c/). Further 4C-seq analyses of our non-coding association regions will allow us to understand which genes or other non-coding regions of DNA these SNPs might be interacting with, and whether the presence of this variation changes these interaction profiles.

### Functional predictions for the novel BMI associated region

Using publicly available data, we compared the novel BMI associated region upstream of IRX5 with gene regulatory markers and functional annotations. This includes (but is not limited to) the presence of CNEs, epigenetic marks and interaction data (HiC). Selecting a relevant cell line is a caveat of this approach, as the exact contribution of this genomic region to BMI is not fully understood, however a recent study suggests the contribution of variation at the FTO locus affects adipocyte lipid accumulation through increased IRX3 and IRX5 expression [40]. Within the novel region there are three CNEs (Additional file 13: Figure S11). These highly constrained regions are strong indicators of regulatory function. One of these CNEs contains, and is surrounded by, a cluster of conserved transcription factor binding sites (HMR Conserved Transcription Factor Binding Sites). In addition, ENCODE Genome Institute of Singapore ChiA-PET data show interactions in our highlighted region for RNAPII and CTCF long-range binding in two different cell lines (K562 myelogenous leukaemia cells and MCF-7 breast cancer cells). CTCF is thought to be a transcriptional regulator [41] and therefore the presence of long-range CTCF-mediated binding in this region suggests a potential role in either repression or activation through DNA looping. The presence of RNAPII mediated looping can also be indicative of enhancer activity in the region. These long-range interactions across the TAD are supported by previous Hi-C data across the whole 2 Mb and suggest that additional regulatory regions might contribute to the gene expression of IRX3 and IRX5 (Additional file 14: Figure S12). Interestingly we are unable to find any positive interaction data between our novel BMI association region and the FTO gene (or any other genes within the TAD) in the current literature.

## Discussion and conclusions

Recently, the selective sequencing of regions of the human genome has been achieved using hybridisation capture approaches. This has largely been exploited to sequence the coding, or exome, portions of the genome. However, the same capture approach can also be adapted to select any regions from the genome [42]. Here, unusually, we have employed it to capture a contiguous megabase scale region of the human genome. The 2 Mb interval was selected using 8,701 probes at intervals of approximately 200 bp. Seventy-two gaps in the sequence, largely repetitive and covering a total of 30.7 kb (1.5 % of total), were not traversable. Between 70 and 80 % of all reads map back to the region of interest in our 284 study group samples providing good coverage across 98.5 % of the interval, making the capture approach considerably more efficient than whole genome sequencing. Furthermore, downstream analysis is considerably simpler and less time-consuming. As a result, we were able to generate very high coverage, (average 200-fold) which in practice means that almost every individual variant can be called with very high confidence.

We decided at the outset to sequence only those individuals that are homozygous at rs9939609. This allowed very high resolution mapping of haplotypes, particularly across the 44 kb LD region associated with this SNP. One of the aims was to determine whether, within the Danish study group we sequenced, there were any low frequency variants that contributed a significant effect within this region and would therefore allow further dissection of the association with BMI. It was also easier to determine whether the association of other variants across the 2 Mb interval with BMI was linked to, or independent of, the 44 kb LD region. Consistent with other studies [43], particularly in European populations, the 44 kb region is in almost complete LD. Incredibly, of 282 SNPs we mapped across the 44 kb, only one (rs16952522:C > G at 53,807,498) is found in common between the rs9939609 ‘A’ and ‘T’ alleles (MAF 0.045 in cases, 0.037 in controls). This implies that at least in the Danish population, recombination events in this region are historically exceptionally rare. The small size of our study group means that we do not have the statistical power to evaluate whether any of the rs9939609 ‘A’ risk allele sub-haplotypes or rare variants are more associated with obesity than others.

Analysis of the constrained sequences within the region confirms that there are no coding variants nor any frequently occurring variants in highly conserved non-coding elements (CNEs) that are associated with elevated BMI. Functional constraint does have an effect on both the frequency of variant locations (4.1, 5.6 and 7.0 per kb, respectively, for coding, CNE and non-coding sequence) and the minor allele frequency of variants (85 % of coding, 80 % of CNE and 70 % of non-coding variants have MAF < 0.1).

Within our Danish male study group, we clearly identify a second, novel region associated with BMI in non-coding sequence upstream of the *IRX5* gene. Individuals in our study group who are homozygous for this second region have a mean BMI elevated to a similar extent to the effect of the known FTO intron region variants. This association is independent of the FTO LD region as it is not present if we compare A/A vs. T/T individuals. Our analysis was performed using data obtained at Danish draft board assessment which results in a very homogeneous study group, not only in terms of gender and ethnicity but also because all participants were of similar age (average age 19.9 years) when their BMI was measured. Interestingly, this association is strongest in younger sub-groups of our replication cohorts as well, suggesting an age-dependence aspect.

To address this idea, we first used imputed values for the three most highly associating variants upstream of *IRX5* in the expanded male cohort, comprising 1,450 individuals, to confirm the association. Next, we used imputed values for the same three SNPs in a completely independent female Danish study group, comprising nearly 4,000 individuals. When we use the entire cohort with a higher average age, the association is not clear but in women aged under 26 years we replicate the association. Thus, if we match the male and female cohorts by age (as far as possible), there remains a significant association between BMI and the region upstream of IRX5.

We then searched for the 22 highest associating SNPs across the second peak of association in the GIANT consortium BMI based anthropometric data for European populations [44]. Of these 22 SNPs, 13 are included in the GIANT dataset, with over 200,000 individuals having data for these variants, yet none of these SNPs are found to have a significant association with obesity. It is impossible to discern the reason for the lack of an association in the GIANT consortium data without secondary analyses. Nevertheless, in our analyses the association is lost as age increases, perhaps because environmental factors such as diet and levels of physical activity are likely to have an increasing impact on BMI with age, confounding the detection of some genetic associations. Conversely, if the genetic consequences of this association are established early in life, such as during development, then it is likely that a stronger association will be seen at a younger age. Given that this locus is intimately associated with complex developmental transcription factors, this would seem highly likely and reflect the life course data at the neighbouring FTO gene [26].

The IRX genes, including *IRX3* and *IRX5*, play complex and overlapping developmental roles in multiple tissues and organs [45, 46]. There is also evidence that both IRX clusters form complex interactions that define specific three-dimensional structures that regulate gene expression at different loci [23, 47]. In particular, it has been shown that the *IRX3* promoter region interacts with a number of distal sites across the 2 Mb region [21] sequenced here and defined by the embryonic stem cell line TAD described previously [24].

In order to examine this in detail, we first looked at the overlap between the interaction data for *FTO* and *IRX3* genes and our association data across the region. As the interaction data are from mouse, we lifted our human data for the region over to the syntenic region on mouse chromosome 8 (methods). There is no strong correlation between *IRX3* (or *FTO*) interactions and either of the BMI-associated regions although there is some *IRX3* and *FTO* signal across the 44 kb region. However, the fact that long-range *IRX3* interactions occur up to and beyond both associated regions suggests architecture is an important aspect of gene regulation across the whole region. This is supported by Hi-C interaction data across the TAD from human embryonic stem cells [24], which clearly show strong interactions between the *FTO* and *IRX5* gene regions. It will therefore be important to establish the specific interaction domains of *IRX5* and *IRX6* in order to get a fuller picture of the complex structure of this region and to be able to place the associated regions into a fuller context.

Despite many GWAS studies [37] and now the full sequencing of this region from a well-defined study group, there remains considerable difficulty in predicting, describing or functionally assaying the impact of non-coding variants on disease or phenotype. As a result, a number of the genes across this region have been implicated in obesity yet without any clear mechanism of regulatory control [48, 49]. This region is particularly complex because of the presence of the IRXB cluster, a set of homologous genes that regulate many aspects of early development and are thus under tight regulatory control themselves. This control is likely to be mediated via cis-regulatory sequences that in some cases may be hundreds of kb away and even within the non-coding regions of other genes, as has been demonstrated for other genes such as *Shh* [50, 51].

The implication of more than one gene in the aetiology of obesity at this locus may therefore not be so surprising, neither is the identification of a second cluster of associating SNPs. The structural architecture(s) of this particular topologically associated domain (TAD) may have profound effects on the regulation of all the genes in the region at some stage, but at this juncture we do not know enough about how sequence variation may alter chromatin architecture nor what the consequences might be in terms of gene expression. Nevertheless, as we gain more insight into the structure and function of the non-coding DNA in this TAD, the complete sequence of the 2 Mb interval from this study group will provide a valuable resource. Furthermore, targeted region sequencing may be of great utility in examining other such complex regions in fine detail in the future.

## Methods

### Sequencing

Samples were curated and individuals were assessed as described previously [28]. Libraries were prepared for sequencing using Illumina Nextera Rapid Capture Custom Enrichment Kit (Cat ID FC-140-1009). The custom kit included 8,701 probes across the 2 Mb region for 288 samples (Project ID 44309). All samples were run on an Illumina HiSeq 2500 at 100 cycle pair end reads. Ninety-six multiplexed samples were run per flow cell with each multiplex being run twice on Rapid Run mode. Samples were de-multiplexed and converted to FASTQ files using Illumina software CASAVA.

### Ethical statement

The study was approved by the regional scientific ethics committee and by the Danish Data Protection Board and fulfilled the Helsinki Declaration.

### Availability of supporting data

Sequence data (reads) will be available through ENA at http://www.ebi.ac.uk/ena. Accession number PRJEB11794. All other data are contained within the paper or supplementary information files. All other data is fully available on request, without restriction.

### Mapping and variant calling

We mapped sequencing data (FASTQ) files to the hg19 assembly of the human genome, the version in human_g1k_v37.fasta file available from the 1000 Genomes Project (ftp://ftp.1000genomes.ebi.ac.uk/vol1/ftp/technical/reference/). We use BWA (Burrows-Wheeler Aligner) software to map the reads [52], version bwa-0.7.8, bwa-mem algorithm with default parameters. The mapped read (sam) files were then converted to bam format using samtools version 0.1.19 [53]. The reads in each bam file were then sorted by chromosome and coordinate and indexed using samtools. Duplicate reads were marked by Picard (http://broadinstitute.github.io/picard), version 1.91, MarkDuplicates tool. Then the two bam files from different sequencing runs for each individual were merged using Picard tool MergeSamFiles. At this point we have one bam file per individual. These bam files were then processed by our in-house tool ‘TidyVar’ (B. Noyvert and G. Elgar, manuscript in preparation), which is an implementation of a novel variant calling algorithm. The algorithm uses string matching approach to detect SNPs and short insertions and deletions, the individual genotypes are assigned using pattern recognition. A single vcf file listing all the variants found in all the individuals is produced. We also scanned our sequencing data for copy-number variations (CNV) by looking for abnormal coverage fluctuations. The detected CNVs were then confirmed by the presence of reads bridging over the suspected region.

### Haplotype analysis

Haplotyping was performed with Haploview [38] using the methods described previously for defining linkage disequilibrium blocks [43]. For this programme, only bi-allelic SNPs were used across the region chr16:53,500,000-55,500,000. Comparisons over each variant over 500 kb were performed and settings altered from default to ignore Hardy-Weinberg *P* values, and to include only individuals with a minimum of 75 % of all SNPs successfully called. Associations of individual variants and haplotypes were produced through Haploview using the case-control allelic chi-squared test with one degree of freedom for the 2 × 2 contingency table of allele counts for reference and non-reference alleles and for case and control separately [54]. The output *P* values of this were used throughout this study.

### Interaction data liftOver

The UCSC genome browser utility liftOver (http://genome.ucsc.edu/cgi-bin/hgLiftOver) was used as the Batch Coordinate Conversion method to transfer SNP hg19 coordinates to mouse genome build mm9 coordinates using default settings. Conversion of 5,842 SNPs was successful with 5,988 SNP locations failing.

### Genotyping and imputation of replication cohorts

Genome-wide genotyping on the Illumina 610 k quad chip was carried out at the Centre National de Genotypage (CNG), Evry, France. We excluded SNPs with minor allele frequency <1 %, >5 % missing genotypes or which failed an exact test of Hardy-Weinberg equilibrium (HWE) in the controls (*P* <10^−7^). We also excluded any individual who did not cluster with the CEU individuals (Utah residents with ancestry from northern and western Europe) in a multidimensional scaling analysis seeded with individuals from the International HapMap release 22, who had >5 % missing data, outlying heterozygosity of >35 % or <30.2 %, genetic duplicates, one of each pair of genetically related individuals, individuals with sex discrepancies and one individual whose genotyping was discordant with a previous project. We carried out imputation to HapMap release 22 (CEU individuals) using Mach 1.0, Markov Chain Haplotyping. This method was used for both the Male and Female GOYA cohorts [31, 39]. Imputed genotypes for the sequenced 284 men (where available) were compared to the sequenced genotypes called by TidyVar and found to be correct 100 % for rs9939609:T > A and 98.3 % correct for the SNPs rs7186407:A > T, rs12598453:C > G and rs12596270:A > G.

### Topological association domain comparison

The –log10(*P* value) for association of each SNP with the case or control cohort was used in preparation of a variable step .wig file with a scale of 0 to 6 and each line to span 1 base. The coordinates for each SNP were converted using UCSC LiftOver from hg19 to hg18 to fit with the original scaffold used for the Hi-C data. The data we have used for the Hi-C tracks are limited to human embryonic stem cells (hESC). Default max (50) and min (10) values were used for the heat map visualisation ([24] and http://yuelab.org/hi-c/).

### Data sources

CNE locations were taken from CONDOR [25]. Exon coordinates were taken from Ensembl Biomart release version 75 [55]. All sequence coordinates in this study are from GRCh37/hg19. 1KG (100 genomes) variant data are taken from the publicly available ‘ALL.chr16.phase1_release_v3.20101123.snps_indels_svs.genotypes.vcf.gz’ VCF file found at ftp://ftp.1000genomes.ebi.ac.uk/vol1/ftp/release/20110521/.

All dbSNP variants are taken from the NCBI publicly available ‘human_9606_b142_GRCh37p13/VCF/All.vcf.gz’ VCF file found at ftp://ftp.ncbi.nlm.nih.gov/snp/organisms/.

Epigenomic data were sourced and visualised through the WashU Epigenome Browser (http://epigenomegateway.wustl.edu/) using ENCODE GIS Chia-Pet publicly available data [56].

## Additional files

Additional file 1: Table S1. Sample IDs, BMIs, ages and case/control classifications for the samples that were included in this study that passed quality control (n = 284). (DOCX 41 kb) Additional file 2: Figure S1. Summary chart of the number of samples where 90 % of bases have coverage of the different bin values. (TIF 1718 kb) Additional file 3: Figure S2. Distribution of SNPs at the 16q12.2 locus that fall within CNEs (CONDOR) associated with the IRXB cluster. Control frequencies are in blue and case frequencies are in red. (TIF 7312 kb) Additional file 4: Figure S3. Distribution of SNPs at the 16q12.2 locus that fall within coding regions. Control frequencies are in blue and case frequencies are in red. (TIF 4857 kb) Additional file 5: Figure S4. Cumulative frequency distribution of variants in CNEs (blue), coding regions (green) and all variants across the region (red). (TIF 1973 kb) Additional file 6: Figure S5. Association plot of the –log10 (*P* value) of all SNPs across the locus with rs9939609:AA. (TIF 1708 kb) Additional file 7: Table S2. All associating haplotypes (*P* value <0.05) from the Haploview LD block definitions with MAF >0.05. (DOCX 24 kb) Additional file 8: Figure S6. As Figure S6 but re-scaled and with the large peak from AH44 removed. Y-axis scale bar is the same as that of Fig. 3 for comparison. Together, Fig. 3 and Figure S6 show that the second peak of association is independent from the AH44 associated haplotype. (TIF 1741 kb) Additional file 9: Figure S7. Plot of the –log10 (*P* value) for association of all SNPs across the locus using a randomly shuffled case and control definition for the whole study group. (TIF 7304 kb) Additional file 10: Figure S8. Allele frequencies by age in Female GOYA cohort. The case allele frequency (AF) is shown in red, the control AF is shown in blue. The allele frequencies are calculated in groups of 400 individuals of consecutive age. While the case AF is consistently larger than control AF for rs9939609:T > A across essentially the entire age range, the consistent AF difference for rs12598453:C > G is only observed in younger (age up to approximately 25 years) women. (TIFF 6788 kb) Additional file 11: Figure S9. Age dependence of SNP rs12598453:C > G association to obesity. Each point on the plot represents the association *P* value (on y-axes) for a subgroup of combined GOYA male and female cohorts younger than a certain age (on x-axes). (TIF 70 kb) Additional file 12: Figure S10. Comparison of our SNP association data with previously published Hi-C data. Our two significant SNP association peaks lie within interacting domains within the previously defined TAD [24]. (TIF 1540 kb) Additional file 13: Figure S11. UCSC browser figure of the second association peak region (54820000-54860000). (TIF 36 kb) Additional file 14: Figure S12. WashU epigenome browser figure. The entire region sequenced is shown. Highlighted in yellow is the second novel peak of association we have identified. (TIF 226 kb)
