# Supplementary material for: Complete re-sequencing of a 2Mb topological domain encompassing the FTO/IRXB genes identifies a novel obesity-associated region upstream of IRX5
Source: Genome Med. 2015 Dec 7;7:126. doi: 10.1186/s13073-015-0250-3 (PMC4671217; doi:10.1186/s13073-015-0250-3)
Supplement: Additional file 1: Table S1. — Sample IDs, BMIs, ages and case/control classifications for the samples that were included in this study that passed quality control (n = 284). (DOCX 41 kb) [file 13073_2015_250_MOESM1_ESM.docx]

**Supplementary Table 1:**

| **IID** | **Case/Control (1/2)** | **Age** | **BMI** |
| --- | --- | --- | --- |
| 3909 | 1 | 19 | 20.9 |
| 4337 | 1 | 22 | 25.5 |
| 4347 | 2 | 19 | 34.0 |
| 4348 | 2 | 26 | 31.6 |
| 4353 | 2 | 19 | 32.4 |
| 4358 | 2 | 19 | 33.6 |
| 4362 | 1 | 19 | 22.3 |
| 4363 | 1 | 24 | 21.2 |
| 4375 | 1 | 24 | 23.9 |
| 4378 | 1 | 21 | 17.1 |
| 4380 | 1 | 19 | 20.6 |
| 4393 | 2 | 19 | 31.7 |
| 4395 | 2 | 19 | 31.9 |
| 4396 | 2 | 19 | 31.9 |
| 4397 | 1 | 18 | 21.1 |
| 4412 | 1 | 19 | 20.7 |
| 4415 | 1 | 23 | 21.1 |
| 4416 | 2 | 19 | 35.7 |
| 4462 | 1 | 19 | 17.8 |
| 4607 | 2 | 19 | 34.5 |
| 4649 | 2 | 20 | 36.7 |
| 4652 | 2 | 19 | 33.9 |
| 4653 | 1 | 19 | 19.8 |
| 4658 | 1 | 20 | 21.4 |
| 4662 | 1 | 20 | 26.2 |
| 4670 | 2 | 20 | 31.1 |
| 4672 | 2 | 19 | 34.2 |
| 4673 | 2 | 18 | 31.6 |
| 4675 | 2 | 19 | 32.7 |
| 4683 | 1 | 19 | 21.3 |
| 4704 | 2 | 22 | 32.8 |
| 4709 | 1 | 19 | 24.0 |
| 4714 | 2 | 20 | 31.2 |
| 4716 | 1 | 20 | 21.3 |
| 4719 | 2 | 24 | 35.4 |
| 4723 | 2 | 19 | 32.8 |
| 4727 | 2 | 19 | 31.6 |
| 4749 | 1 | 18 | 20.7 |
| 4751 | 1 | 19 | 21.4 |
| 4755 | 1 | 19 | 21.5 |
| 4772 | 1 | 18 | 19.9 |
| 4776 | 1 | 19 | 20.2 |
| 4779 | 1 | 19 | 18.9 |
| 4780 | 2 | 20 | 31.6 |
| 4781 | 1 | 19 | 17.8 |
| 4782 | 1 | 19 | 24.5 |
| 4792 | 1 | 25 | 19.6 |
| 4798 | 1 | 20 | 21.1 |
| 4826 | 2 | 21 | 32.5 |
| 4846 | 2 | 19 | 31.6 |
| 4860 | 1 | 19 | 21.7 |
| 4990 | 2 | 19 | 32.0 |
| 5301 | 1 | 20 | 20.3 |
| 5305 | 1 | 18 | 21.8 |
| 5308 | 2 | 19 | 32.7 |
| 5370 | 1 | 23 | 22.0 |
| 5373 | 1 | 19 | 21.4 |
| 5374 | 1 | 19 | 21.3 |
| 5397 | 2 | 18 | 33.0 |
| 5399 | 2 | 20 | 33.8 |
| 5407 | 2 | 19 | 33.5 |
| 5444 | 1 | 19 | 22.3 |
| 5469 | 1 | 20 | 20.7 |
| 5477 | 1 | 19 | 21.6 |
| 5479 | 2 | 19 | 32.1 |
| 5481 | 1 | 20 | 20.7 |
| 5482 | 2 | 20 | 32.1 |
| 5508 | 1 | 20 | 21.1 |
| 5525 | 2 | 20 | 37.4 |
| 5533 | 1 | 21 | 18.3 |
| 5550 | 1 | 20 | 20.7 |
| 5553 | 1 | 18 | 29.7 |
| 5583 | 1 | 26 | 20.9 |
| 5623 | 1 | 20 | 22.4 |
| 5695 | 2 | 20 | 32.0 |
| 5706 | 2 | 19 | 32.0 |
| 5901 | 1 | 18 | 23.6 |
| 6001 | 2 | 19 | 34.6 |
| 6884 | 2 | 20 | 32.8 |
| 6898 | 1 | 18 | 24.3 |
| 6903 | 2 | 19 | 33.1 |
| 6906 | 1 | 19 | 25.9 |
| 6908 | 1 | 19 | 23.3 |
| 6910 | 1 | 18 | 23.8 |
| 6923 | 2 | 21 | 31.8 |
| 6924 | 2 | 19 | 35.3 |
| 6932 | 1 | 20 | 21.1 |
| 6934 | 1 | 19 | 18.6 |
| 6964 | 2 | 18 | 36.3 |
| 6976 | 1 | 21 | 20.4 |
| 6981 | 1 | 20 | 21.2 |
| 7088 | 1 | 19 | 20.5 |
| 7215 | 2 | 19 | 31.5 |
| 7257 | 2 | 19 | 31.1 |
| 7269 | 1 | 18 | 26.3 |
| 7277 | 2 | 19 | 31.1 |
| 7294 | 2 | 19 | 31.2 |
| 7368 | 1 | 20 | 21.9 |
| 7404 | 1 | 19 | 19.8 |
| 7406 | 1 | 19 | 21.4 |
| 7408 | 1 | 20 | 24.8 |
| 7593 | 1 | 19 | 22.3 |
| 7647 | 1 | 28 | 25.1 |
| 7653 | 1 | 19 | 23.2 |
| 7928 | 2 | 19 | 32.8 |
| 7941 | 2 | 18 | 33.9 |
| 8048 | 1 | 19 | 20.0 |
| 8051 | 2 | 19 | 33.3 |
| 8072 | 2 | 19 | 33.6 |
| 8155 | 1 | 19 | 18.4 |
| 8277 | 2 | 18 | 31.2 |
| 8289 | 1 | 19 | 21.2 |
| 8501 | 1 | 20 | 22.9 |
| 8552 | 2 | 19 | 31.8 |
| 8553 | 1 | 20 | 22.6 |
| 8555 | 1 | 19 | 17.2 |
| 8560 | 2 | 19 | 43.3 |
| 8726 | 1 | 21 | 17.8 |
| 8792 | 2 | 25 | 33.0 |
| 8807 | 1 | 19 | 17.9 |
| 8880 | 1 | 21 | 24.2 |
| 8882 | 1 | 21 | 24.3 |
| 8921 | 1 | 18 | 20.9 |
| 8928 | 2 | 19 | 35.3 |
| 8934 | 2 | 19 | 32.5 |
| 8994 | 1 | 18 | 25.6 |
| 9016 | 1 | 19 | 18.9 |
| 9017 | 2 | 19 | 32.3 |
| 9075 | 1 | 19 | 20.0 |
| 9078 | 1 | 22 | 23.1 |
| 9108 | 2 | 19 | 51.8 |
| 9424 | 1 | 20 | 20.3 |
| 9472 | 2 | 19 | 31.5 |
| 9664 | 1 | 18 | 20.9 |
| 9769 | 2 | 19 | 31.5 |
| 9785 | 1 | 19 | 22.3 |
| 9789 | 1 | 20 | 19.3 |
| 9805 | 2 | 19 | 34.5 |
| 9808 | 1 | 19 | 24.2 |
| 9882 | 2 | 23 | 32.7 |
| 9913 | 2 | 19 | 33.3 |
| 9924 | 1 | 24 | 20.7 |
| 9957 | 1 | 19 | 18.0 |
| 10577 | 2 | 19 | 35.6 |
| 10600 | 2 | 20 | 33.8 |
| 10645 | 2 | 18 | 40.4 |
| 10713 | 1 | 19 | 21.7 |
| 10741 | 1 | 21 | 20.3 |
| 10769 | 2 | 20 | 34.3 |
| 10811 | 1 | 20 | 17.4 |
| 10835 | 1 | 18 | 20.9 |
| 10858 | 1 | 26 | 20.4 |
| 10875 | 1 | 18 | 19.7 |
| 10878 | 1 | 19 | 17.8 |
| 10881 | 2 | 19 | 31.4 |
| 10885 | 1 | 18 | 21.5 |
| 10886 | 1 | 18 | 26.2 |
| 10889 | 1 | 19 | 19.1 |
| 10898 | 2 | 21 | 31.4 |
| 10899 | 1 | 19 | 21.0 |
| 10905 | 2 | 19 | 31.4 |
| 10911 | 1 | 19 | 22.4 |
| 10912 | 1 | 20 | 22.9 |
| 10913 | 1 | 21 | 28.1 |
| 10915 | 2 | 19 | 31.7 |
| 10917 | 1 | 18 | 20.3 |
| 10942 | 1 | 18 | 22.1 |
| 10944 | 1 | 19 | 18.6 |
| 10948 | 1 | 19 | 19.4 |
| 10957 | 2 | 19 | 32.7 |
| 10971 | 2 | 19 | 35.1 |
| 10978 | 1 | 20 | 21.4 |
| 10982 | 1 | 19 | 19.4 |
| 10986 | 1 | 19 | 20.8 |
| 10989 | 2 | 20 | 33.0 |
| 11006 | 1 | 20 | 19.2 |
| 11012 | 1 | 20 | 21.8 |
| 11022 | 2 | 19 | 31.5 |
| 11025 | 1 | 21 | 25.6 |
| 11059 | 2 | 20 | 36.2 |
| 11065 | 2 | 19 | 36.6 |
| 11074 | 1 | 20 | 18.8 |
| 11078 | 1 | 19 | 18.2 |
| 11082 | 1 | 19 | 22.9 |
| 11089 | 2 | 19 | 31.3 |
| 11093 | 2 | 18 | 32.1 |
| 11096 | 1 | 21 | 21.5 |
| 11101 | 1 | 20 | 19.3 |
| 11103 | 2 | 19 | 31.5 |
| 11115 | 1 | 26 | 22.9 |
| 11124 | 1 | 20 | 19.8 |
| 11129 | 1 | 19 | 21.7 |
| 11135 | 1 | 20 | 19.6 |
| 11141 | 2 | 19 | 32.2 |
| 11145 | 1 | 20 | 24.8 |
| 11164 | 1 | 19 | 22.3 |
| 11167 | 1 | 20 | 21.1 |
| 11176 | 2 | 18 | 31.3 |
| 11183 | 1 | 19 | 19.1 |
| 11189 | 1 | 19 | 18.6 |
| 11205 | 1 | 19 | 22.1 |
| 11214 | 2 | 19 | 31.8 |
| 11234 | 2 | 21 | 31.0 |
| 11239 | 1 | 19 | 20.8 |
| 11240 | 2 | 22 | 31.1 |
| 11266 | 1 | 19 | 19.1 |
| 11276 | 1 | 19 | 15.9 |
| 11277 | 1 | 26 | 22.3 |
| 11286 | 2 | 20 | 31.4 |
| 11287 | 1 | 19 | 23.1 |
| 11297 | 2 | 19 | 34.0 |
| 11304 | 2 | 19 | 32.1 |
| 11309 | 2 | 19 | 34.8 |
| 11323 | 2 | 26 | 32.1 |
| 11324 | 2 | 20 | 35.0 |
| 11332 | 2 | 19 | 32.5 |
| 11334 | 1 | 20 | 22.4 |
| 11345 | 1 | 19 | 20.8 |
| 11348 | 1 | 19 | 21.0 |
| 11349 | 1 | 21 | 25.1 |
| 11350 | 2 | 27 | 35.2 |
| 11353 | 1 | 18 | 18.8 |
| 11356 | 1 | 21 | 26.6 |
| 11359 | 2 | 19 | 35.0 |
| 11373 | 1 | 19 | 24.4 |
| 11381 | 2 | 19 | 35.0 |
| 11383 | 1 | 19 | 24.3 |
| 11384 | 1 | 31 | 23.4 |
| 11399 | 1 | 18 | 22.9 |
| 11402 | 2 | 18 | 31.1 |
| 11408 | 1 | 19 | 22.5 |
| 11410 | 2 | 20 | 33.0 |
| 11416 | 1 | 19 | 19.6 |
| 11429 | 2 | 25 | 31.1 |
| 11439 | 2 | 20 | 36.2 |
| 11440 | 2 | 19 | 31.2 |
| 11442 | 2 | 19 | 37.9 |
| 11448 | 1 | 20 | 19.3 |
| 11453 | 2 | 18 | 31.3 |
| 11459 | 2 | 18 | 33.5 |
| 11478 | 2 | 19 | 35.4 |
| 11481 | 2 | 21 | 34.7 |
| 11497 | 2 | 19 | 32.0 |
| 11500 | 2 | 19 | 36.2 |
| 11507 | 2 | 19 | 31.6 |
| 11509 | 2 | 19 | 33.2 |
| 11512 | 2 | 19 | 32.5 |
| 11515 | 1 | 19 | 20.5 |
| 11519 | 1 | 21 | 23.8 |
| 11533 | 1 | 22 | 24.6 |
| 11535 | 2 | 26 | 31.6 |
| 11538 | 1 | 19 | 22.0 |
| 11555 | 2 | 19 | 31.5 |
| 11568 | 1 | 19 | 22.9 |
| 11570 | 2 | 19 | 31.7 |
| 11585 | 1 | 22 | 24.8 |
| 11586 | 2 | 19 | 31.6 |
| 11595 | 2 | 19 | 33.8 |
| 11602 | 1 | 25 | 19.3 |
| 11616 | 2 | 20 | 31.1 |
| 11623 | 1 | 20 | 19.8 |
| 11631 | 1 | 24 | 26.5 |
| 11642 | 2 | 19 | 31.2 |
| 11643 | 2 | 20 | 31.2 |
| 11655 | 2 | 21 | 32.1 |
| 11660 | 2 | 19 | 33.3 |
| 11666 | 1 | 23 | 25.0 |
| 11668 | 1 | 19 | 21.6 |
| 11672 | 2 | 20 | 31.3 |
| 11673 | 2 | 19 | 31.1 |
| 11675 | 2 | 19 | 32.3 |
| 11681 | 1 | 20 | 21.7 |
| 11688 | 2 | 19 | 32.7 |
| 11727 | 2 | 18 | 31.6 |
| 11736 | 2 | 19 | 32.5 |
| 11741 | 1 | 18 | 20.2 |
| 11753 | 1 | 23 | 18.7 |
| 11755 | 1 | 19 | 18.8 |
| 11756 | 1 | 20 | 21.4 |
| 11762 | 1 | 18 | 23.1 |
| 11763 | 1 | 20 | 19.8 |
| 11774 | 1 | 19 | 23.1 |
| 11779 | 1 | 20 | 19.2 |
| 11780 | 1 | 19 | 22.5 |
