# Supplementary figures and images for: Complete re-sequencing of a 2Mb topological domain encompassing the FTO/IRXB genes identifies a novel obesity-associated region upstream of IRX5
Source: Genome Med. 2015 Dec 7;7:126. doi: 10.1186/s13073-015-0250-3 (PMC4671217; doi:10.1186/s13073-015-0250-3)

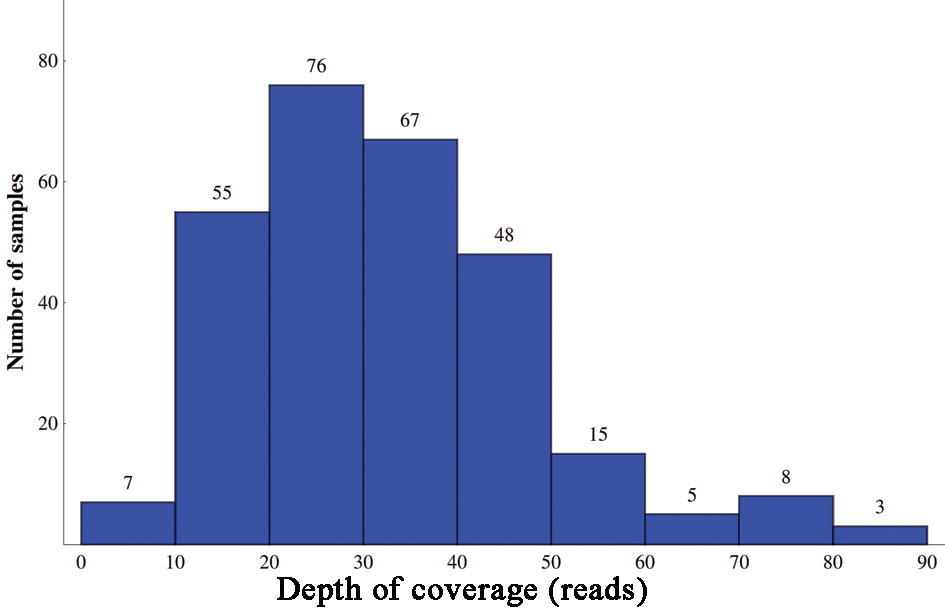

Supplement: Additional file 2: Figure S1. — Summary chart of the number of samples where 90 % of bases have coverage of the different bin values. (TIF 1718 kb) [file 13073_2015_250_MOESM2_ESM.tif]

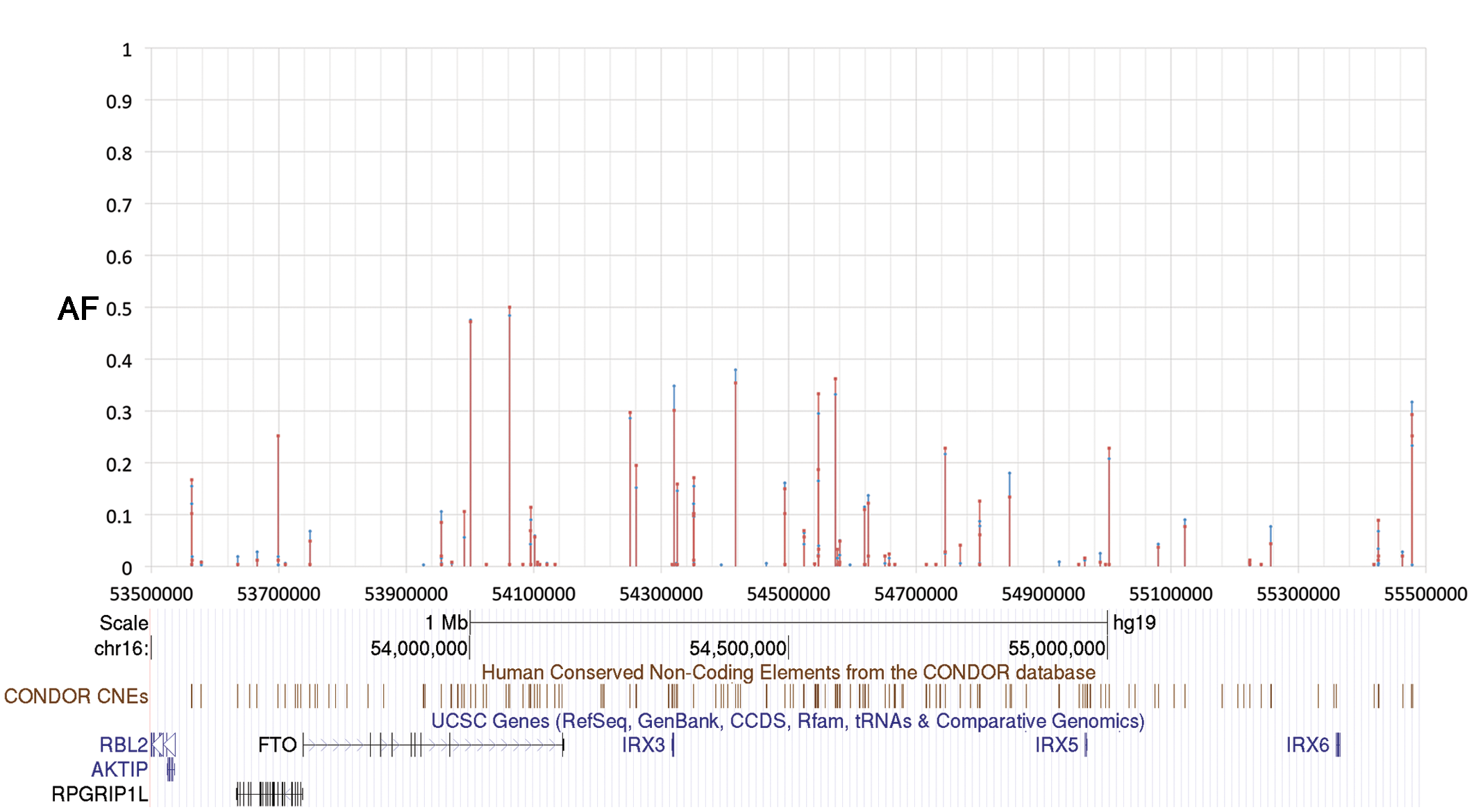

Supplement: Additional file 3: Figure S2. — Distribution of SNPs at the 16q12.2 locus that fall within CNEs (CONDOR) associated with the IRXB cluster. Control frequencies are in blue and case frequencies are in red. (TIF 7312 kb) [file 13073_2015_250_MOESM3_ESM.tif]

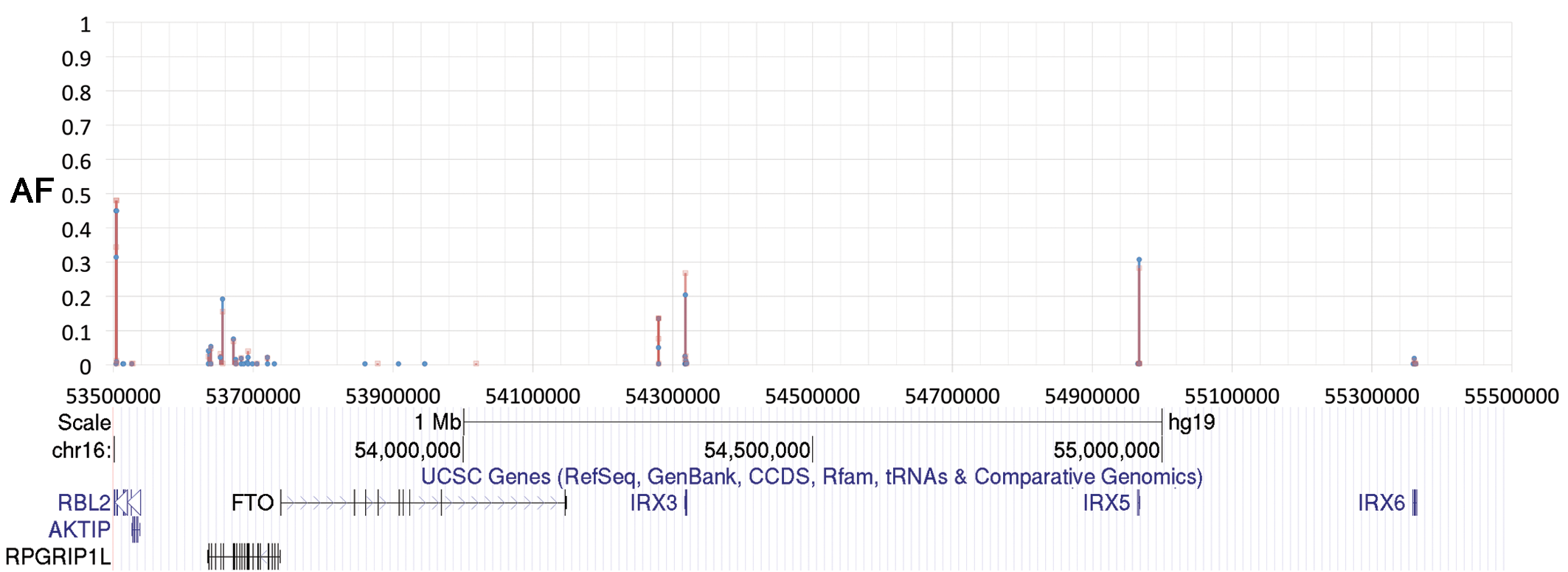

Supplement: Additional file 4: Figure S3. — Distribution of SNPs at the 16q12.2 locus that fall within coding regions. Control frequencies are in blue and case frequencies are in red. (TIF 4857 kb) [file 13073_2015_250_MOESM4_ESM.tif]

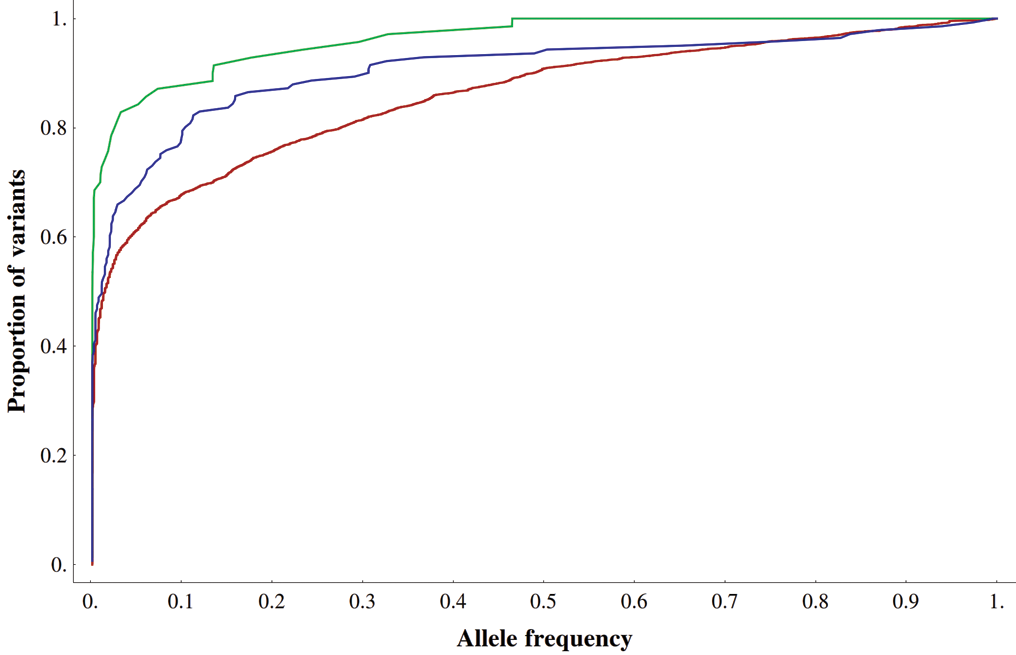

Supplement: Additional file 5: Figure S4. — Cumulative frequency distribution of variants in CNEs (blue), coding regions (green) and all variants across the region (red). (TIF 1973 kb) [file 13073_2015_250_MOESM5_ESM.tif]

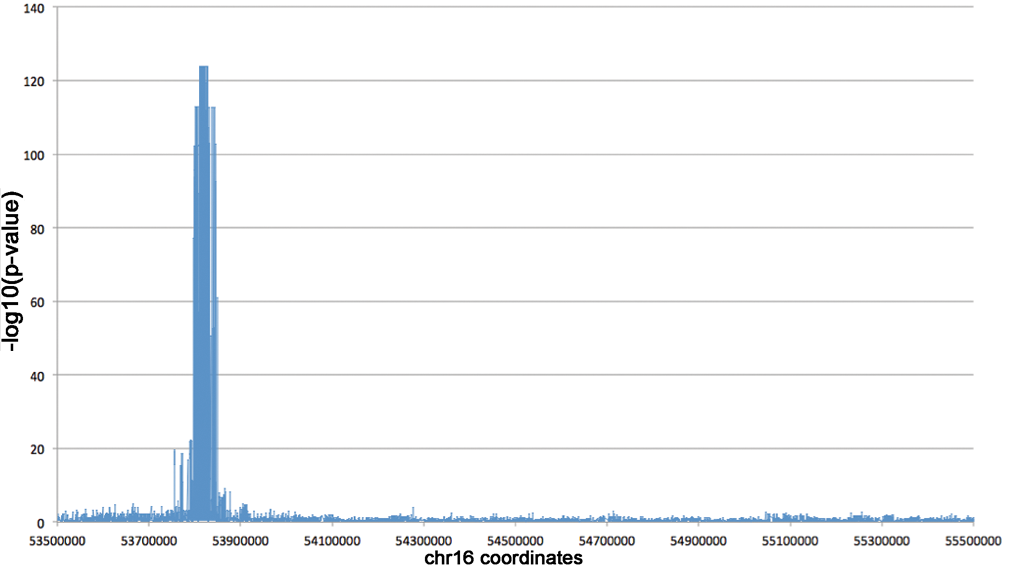

Supplement: Additional file 6: Figure S5. — Association plot of the –log10 (P value) of all SNPs across the locus with rs9939609:AA. (TIF 1708 kb) [file 13073_2015_250_MOESM6_ESM.tif]

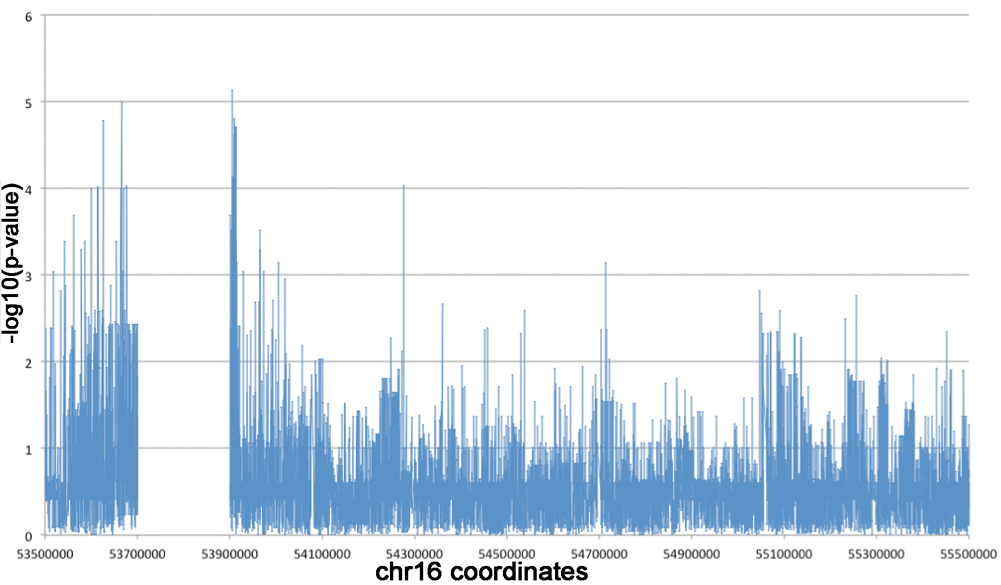

Supplement: Additional file 8: Figure S6. — As Figure S6 but re-scaled and with the large peak from AH44 removed. Y-axis scale bar is the same as that of Fig. 3 for comparison. Together, Fig. 3 and Figure S6 show that the second peak of association is independent from the AH44 associated haplotype. (TIF 1741 kb) [file 13073_2015_250_MOESM8_ESM.tif]

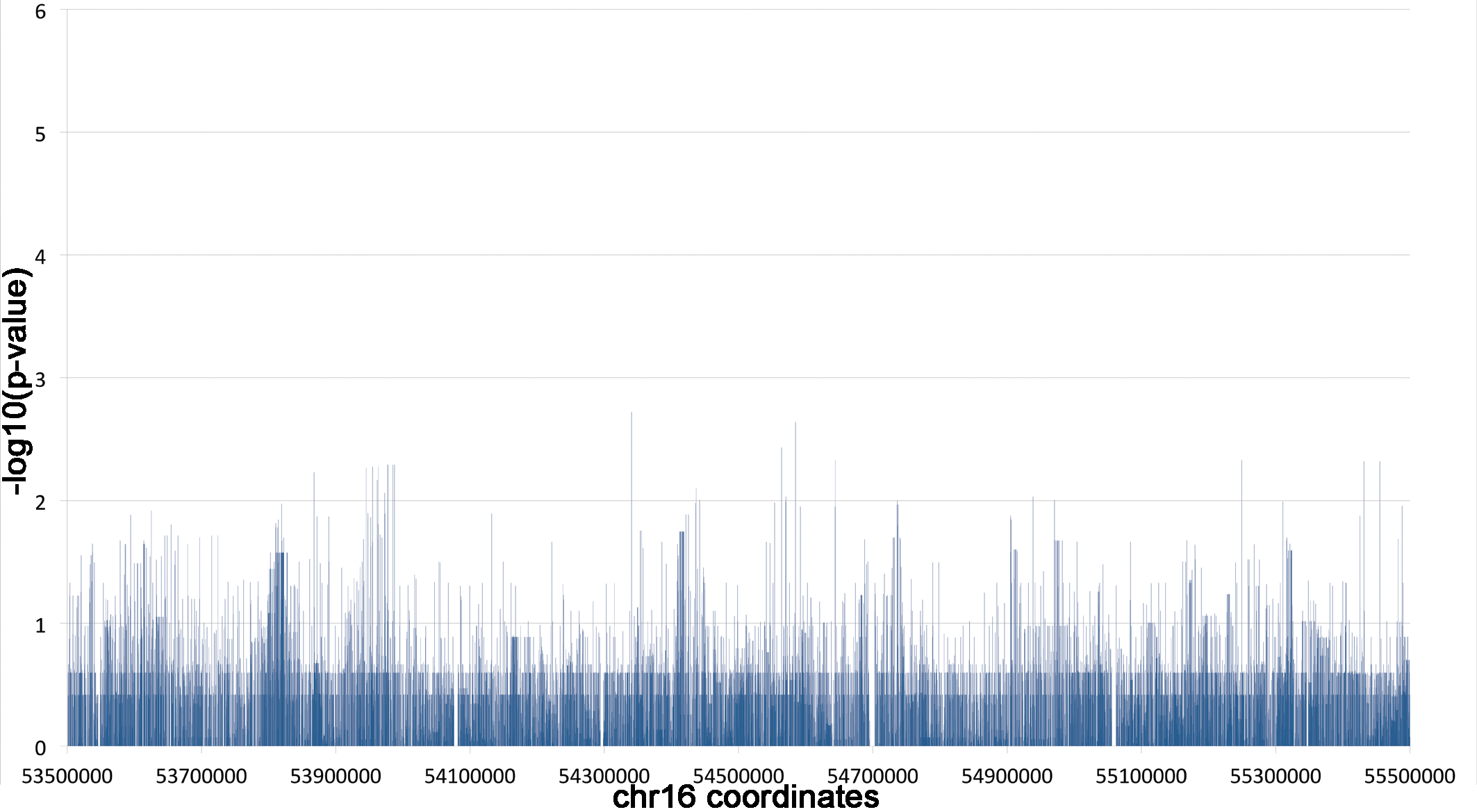

Supplement: Additional file 9: Figure S7. — Plot of the –log10 (P value) for association of all SNPs across the locus using a randomly shuffled case and control definition for the whole study group. (TIF 7304 kb) [file 13073_2015_250_MOESM9_ESM.tif]

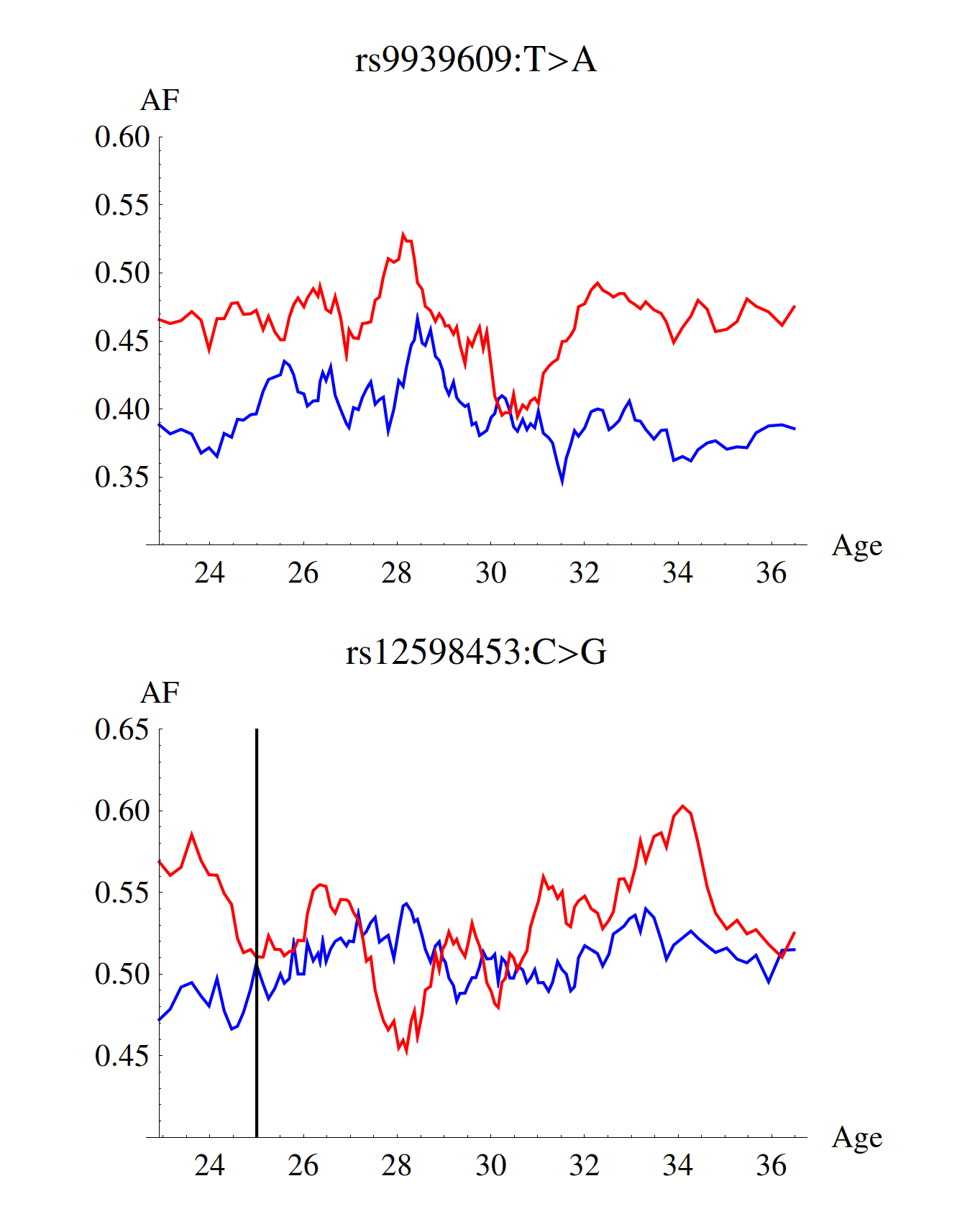

Supplement: Additional file 10: Figure S8. — Allele frequencies by age in Female GOYA cohort. The case allele frequency (AF) is shown in red, the control AF is shown in blue. The allele frequencies are calculated in groups of 400 individuals of consecutive age. While the case AF is consistently larger than control AF for rs9939609:T > A across essentially the entire age range, the consistent AF difference for rs12598453:C > G is only observed in younger (age up to approximately 25 years) women. (TIFF 6788 kb) [file 13073_2015_250_MOESM10_ESM.tiff]

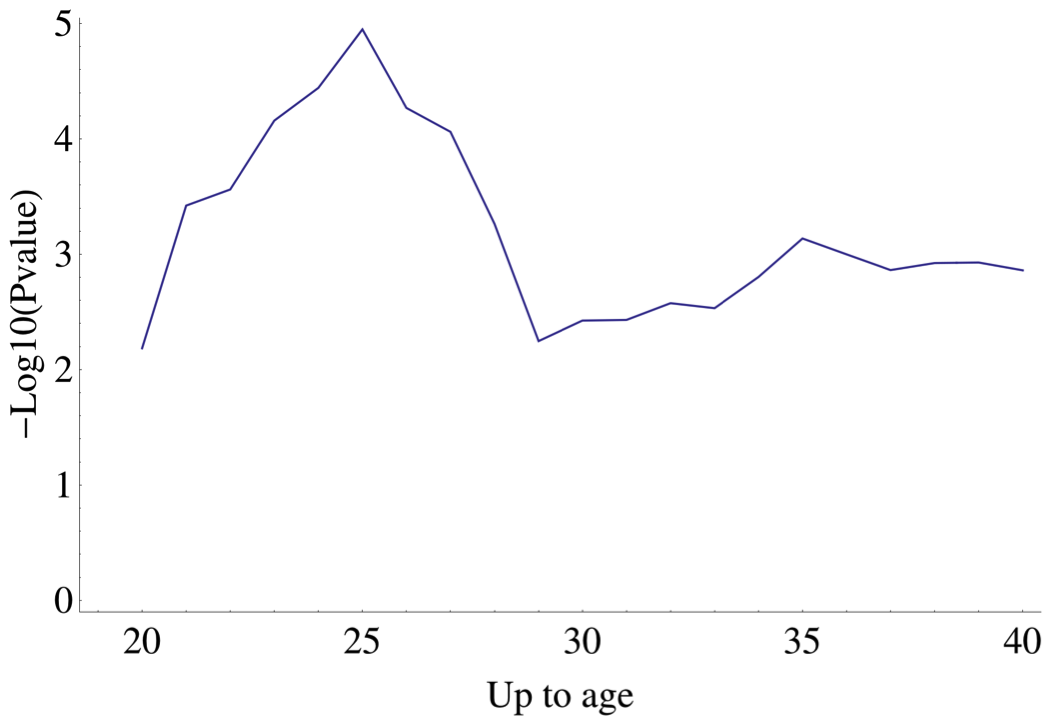

Supplement: Additional file 11: Figure S9. — Age dependence of SNP rs12598453:C > G association to obesity. Each point on the plot represents the association P value (on y-axes) for a subgroup of combined GOYA male and female cohorts younger than a certain age (on x-axes). (TIF 70 kb) [file 13073_2015_250_MOESM11_ESM.tif]

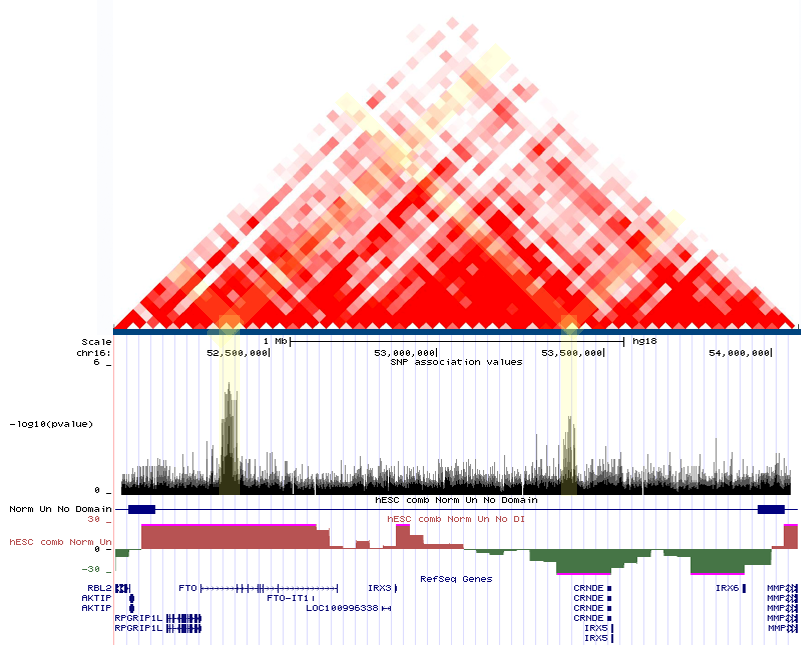

Supplement: Additional file 12: Figure S10. — Comparison of our SNP association data with previously published Hi-C data. Our two significant SNP association peaks lie within interacting domains within the previously defined TAD [24]. (TIF 1540 kb) [file 13073_2015_250_MOESM12_ESM.tif]

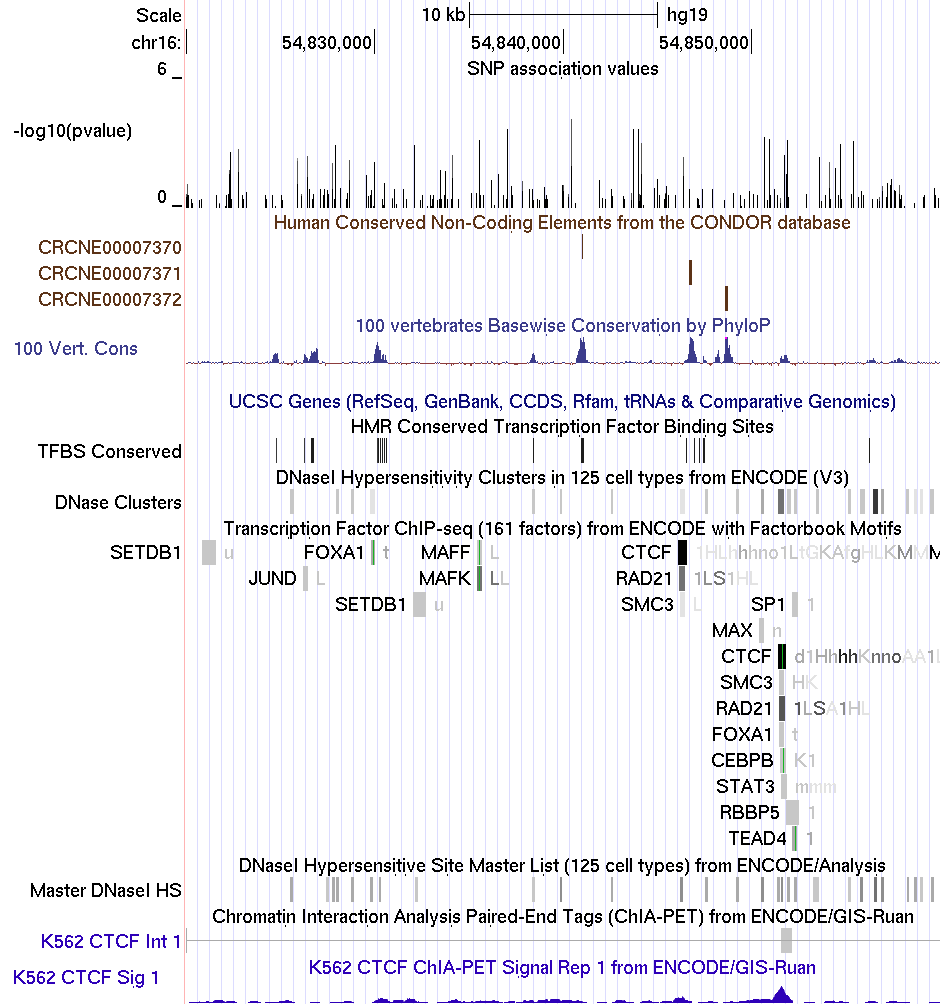

Supplement: Additional file 13: Figure S11. — UCSC browser figure of the second association peak region (54820000-54860000). (TIF 36 kb) [file 13073_2015_250_MOESM13_ESM.tif]

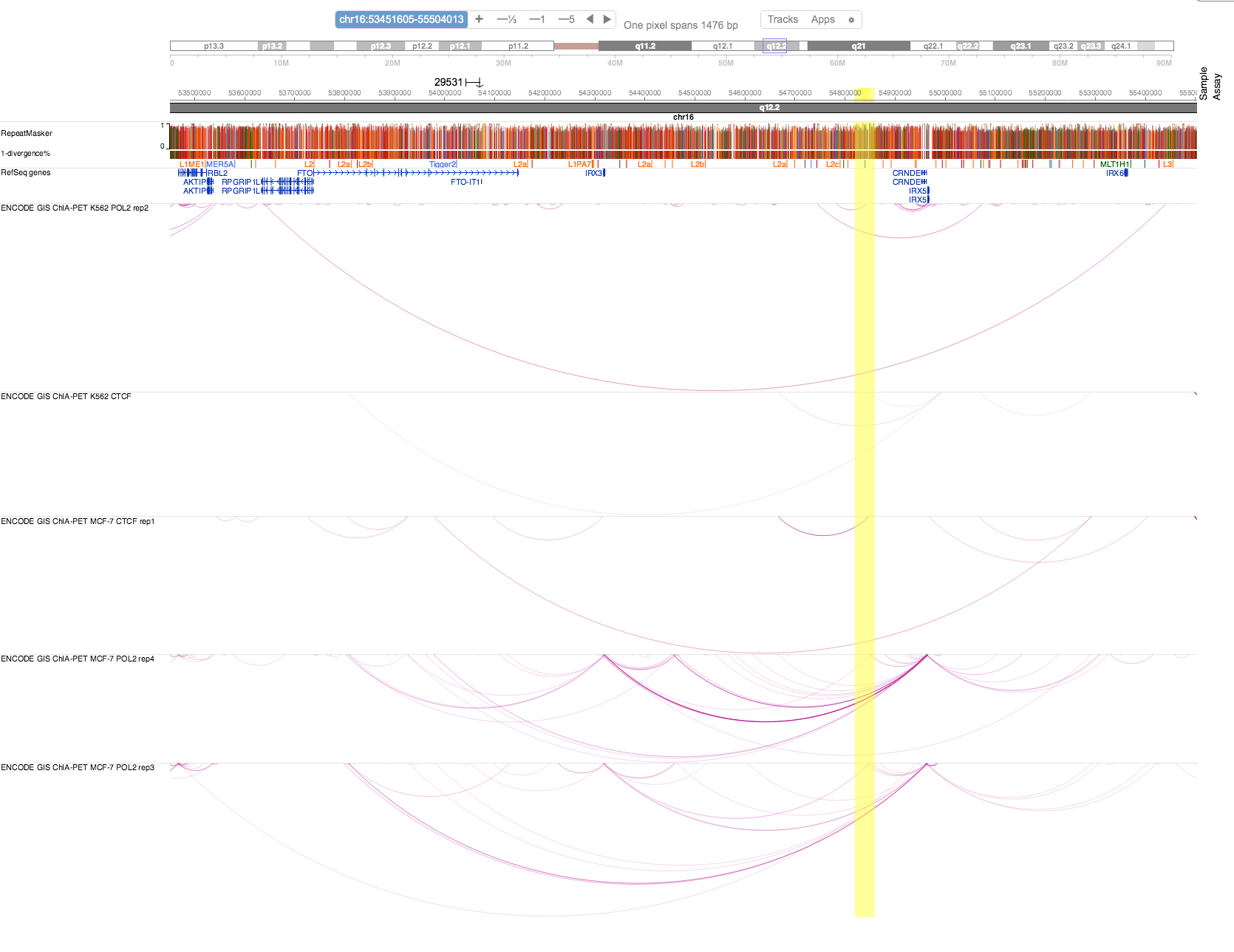

Supplement: Additional file 14: Figure S12. — WashU epigenome browser figure. The entire region sequenced is shown. Highlighted in yellow is the second novel peak of association we have identified. (TIF 226 kb) [file 13073_2015_250_MOESM14_ESM.tif]
