# Supplementary material for: Complete re-sequencing of a 2Mb topological domain encompassing the FTO/IRXB genes identifies a novel obesity-associated region upstream of IRX5
Source: Genome Med. 2015 Dec 7;7:126. doi: 10.1186/s13073-015-0250-3 (PMC4671217; doi:10.1186/s13073-015-0250-3)
Supplement: Additional file 7: Table S2. — All associating haplotypes (P value <0.05) from the Haploview LD block definitions with MAF >0.05. (DOCX 24 kb) [file 13073_2015_250_MOESM7_ESM.docx]

**Supplementary Table 2.**

| **Start** | **End** | **Tagged SNP region** | **Haplotype** | **Freq.** | **Case, Control Frequencies** | **P Value** |
| --- | --- | --- | --- | --- | --- | --- |
| **53606229** | **53739773** | **587-1267** | **Block 9** |  |  |  |
|  |  |  | GAAGAACCTATGGGCTTCCTGTGTGCTCGATTTTCCAACCACCGGGCGTTCAGCTTCCTGATCCCATCGCTCGAGTGCGTCCACCTCCAGGCGCTAGAACTTTGACTCAGAAATCAACCTCCAAAAGATCTGCATAAA | 0.264 | 0.315, 0.233 | 0.0327 |
| **53755146** | **53759123** | **1330-1349** | **Block 12** |  |  |  |
|  |  |  | GGTCTAGC | 0.516 | 0.577, 0.469 | 0.0105 |
|  |  |  | AACTCAGC | 0.313 | 0.260, 0.354 | 0.0169 |
| **53767959** | **53771583** | **1379-1398** | **Block 13** |  |  |  |
|  |  |  | CAAAAAAGT | 0.523 | 0.585, 0.475 | 0.0092 |
|  |  |  | CAAGGACGT | 0.299 | 0.252, 0.335 | 0.0316 |
| **53772346** | **53772626** | **1406-1407** | **Block 14** |  |  |  |
|  |  |  | GA | 0.525 | 0.589, 0.475 | 0.0069 |
|  |  |  | AT | 0.475 | 0.411, 0.525 | 0.0069 |
| **53774903** | **53786446** | **1427-1469** | **Block 16** |  |  |  |
|  |  |  | ACGAGCGTACT | 0.396 | 0.451, 0.354 | 0.019 |
|  |  |  | ACGAGCGTGCT | 0.375 | 0.325, 0.413 | 0.0321 |
| **53793798** | **53795636** | **1510-1526** | **Block 18** |  |  |  |
|  |  |  | CCGCCGCCGGA | 0.456 | 0.504, 0.419 | 0.0442 |
| **53798523** | **53798622** | **1542-1543** | **Block 20** |  |  |  |
|  |  |  | AT | 0.482 | 0.556, 0.424 | 0.0018 |
|  |  |  | GG | 0.477 | 0.419, 0.522 | 0.0148 |
| **53799296** | **53843533** | **1546-1827** | **Block 21** |  |  |  |
|  |  |  | TTAGGATCAGACATTTATAATCACCGGGGTAACTGCAAGTTCGCACGAGCTTAGGCCAATGTCAAGGCCGGTATGGTAGGACGTCCGGGTGTGTGGCACTTTGCATGCTGCGGGAT | 0.293 | 0.384, 0.232 | 0.0001 |
| **53844579** | **53845487** | **1834-1841** | **Block 22** |  |  |  |
|  |  |  | GTC | 0.439 | 0.362, 0.497 | 0.0013 |
|  |  |  | GGA | 0.434 | 0.532, 0.360 | 0.000041 |
| **54010398** | **54019686** | **2802-2844** | **Block 43** |  |  |  |
|  |  |  | GACACGTCG | 0.198 | 0.150, 0.235 | 0.0114 |
| **54211937** | **54213893** | **4008-4016** | **Block 65** |  |  |  |
|  |  |  | CAGGTCG | 0.29 | 0.340, 0.251 | 0.0211 |
| **54214069** | **54214702** | **4018-4023** | **Block 66** |  |  |  |
|  |  |  | TGCT | 0.289 | 0.337, 0.252 | 0.0253 |
| **54268659** | **54271085** | **4349-4366** | **Block 75** |  |  |  |
|  |  |  | CCTCCC | 0.177 | 0.228, 0.138 | 0.0051 |
| **54272047** | **54306215** | **4371-4583** | **Block 77** |  |  |  |
|  |  |  | CAGATGTTGACGCGGGCATGTGGTACGACTGGCTTGCGTTCGCTCGTCGCGAGAGGGGCGCGTCCCGCTCCGACTCTTCAGATCCGACCCGCCCCGAGTTCACTACTGCACGCCG | 0.054 | 0.082, 0.034 | 0.0143 |
| **54327852** | **54328675** | **4707-4712** | **Block 81** |  |  |  |
|  |  |  | CTTGA | 0.067 | 0.093, 0.047 | 0.0266 |
| **54532641** | **54537608** | **6137-6157** | **Block 115** |  |  |  |
|  |  |  | CCGAT | 0.06 | 0.089, 0.037 | 0.0094 |
| **54542033** | **54546604** | **6183-6198** | **Block 116** |  |  |  |
|  |  |  | ATTCCCGCCC | 0.468 | 0.419, 0.506 | 0.0384 |
| **54736811** | **54741807** | **7291-7311** | **Block 136** |  |  |  |
|  |  |  | TGATCGAAA | 0.202 | 0.158, 0.236 | 0.0216 |
| **54753168** | **54774171** | **7389-7529** | **Block 142** |  |  |  |
|  |  |  | TCGGCGACTCCAAGCCGGATCAACTAAATTGCCGCCTCGGGGATCACCCTAGGGCGAGACAACAACTTGCCACAAGAA | 0.294 | 0.248, 0.330 | 0.0351 |
| **54777074** | **54807769** | **7565-7775** | **Block 144** |  |  |  |
|  |  |  | CGTGGCCAAACGAGTATCAGTGATGAACCGGCCACCTCTC | 0.206 | 0.254, 0.171 | 0.016 |
| **54808449** | **54813519** | **7780-7809** | **Block 145** |  |  |  |
|  |  |  | GTCAGG | 0.295 | 0.356, 0.248 | 0.0055 |
| **54813801** | **54817371** | **7812-7842** | **Block 146** |  |  |  |
|  |  |  | ACCTGAGGC | 0.528 | 0.598, 0.475 | 0.0038 |
| **54818762** | **54856786** | **7850-8069** | **Block 147** |  |  |  |
|  |  |  | TCCTCAGAACCCCCGCGAACCGGCCAGCGCGTCGCAGTTCGACTTGCGGGTAATATAACCGACCCTGCTCATGATCCG | 0.412 | 0.493, 0.362 | 0.0021 |
|  |  |  | TCCTCATAACCCCCCGTAACCGATCAGAGCGTTAGTAATCGACTGGCGCACAATGTAACCGGAAGCGCCCTCCAACCG | 0.125 | 0.089, 0.156 | 0.019 |
| **54856933** | **54857871** | **8071-8080** | **Block 148** |  |  |  |
|  |  |  | TGCGCGA | 0.584 | 0.638, 0.543 | 0.0232 |
| **54885318** | **54963258** | **8251-8666** | **Block 152** |  |  |  |
|  |  |  | AGACTTAACCCCGTATGCGAATACACCAATCGACCAACAGAATGGTTTAGACGAGGCTCTCGTAAATATATTCTCAAGGGTTTATACTCGGACC | 0.132 | 0.167, 0.109 | 0.0466 |
|  |  |  | GAGTCACGTCTTCCGTATCGGCGGCTAGGGAAGTTGTTGCCGTCAGCCTTGTAGAACGTCCACGACGGCGCTCCAGGGAGCCAGCGTCTCATCG | 0.051 | 0.075, 0.035 | 0.0382 |
| **55136446** | **55139619** | **9771-9792** | **Block 181** |  |  |  |
|  |  |  | AAATCAGCAG | 0.107 | 0.077, 0.130 | 0.0424 |
| **55140657** | **55141022** | **9799-9802** | **Block 182** |  |  |  |
|  |  |  | TA | 0.884 | 0.915, 0.860 | 0.0451 |
|  |  |  | CT | 0.116 | 0.085, 0.140 | 0.0451 |
| **55226745** | **55234048** | **10439-10508** | **Block 198** |  |  |  |
|  |  |  | CAACTCGTCCTCCGCTTCGACCAGAGGGGGTCGACATCAAGAA | 0.085 | 0.118, 0.059 | 0.0124 |
| **55234072** | **55276574** | **10509-10821** | **Block 199** |  |  |  |
|  |  |  | CATTGTTGAGCCGTTAGAGGAGGAATCTTTAGAAAGGGCGGCGACGCAGAAGGTCGTTAGGGCGAAAGCATCCGTCAAAAGCTTGCTATTCTGTGTTAGTTCCGTCAGATGATGGCTGCTCCGGGCGTGTTCGCCGTGCAAATATGAGCACTATCTTCACATTTGCGCGGAGGCGG | 0.1 | 0.131, 0.079 | 0.0479 |
| **55279211** | **55291144** | **10839-10899** | **Block 201** |  |  |  |
|  |  |  | ATGAGTGCGACTGAT | 0.13 | 0.167, 0.102 | 0.0243 |
| **55461759** | **55461854** | **11927-11930** | **Block 221** |  |  |  |
|  |  |  | GGT | 0.655 | 0.703, 0.618 | 0.0342 |
